# Phylogeny of Nitrogenase Structural and Assembly Components Reveals New Insights into the Origin and Distribution of Nitrogen Fixation across Bacteria and Archaea

**DOI:** 10.3390/microorganisms9081662

**Published:** 2021-08-04

**Authors:** Amrit Koirala, Volker S. Brözel

**Affiliations:** 1 Department of Biology and Microbiology, South Dakota State University, Brookings, SD 57006, USA; Amrit.koirala@sdstate.edu; 2 Department of Biochemistry, Genetics and Microbiology, University of Pretoria, Pretoria 0004, South Africa

**Keywords:** nitrogenase, diazotroph, horizontal gene transfer, niche, *nifH*, NifHDKENB

## Abstract

The phylogeny of nitrogenase has only been analyzed using the structural proteins NifHDK. As *nifHDKENB* has been established as the minimum number of genes necessary for in silico prediction of diazotrophy, we present an updated phylogeny of diazotrophs using both structural (NifHDK) and cofactor assembly proteins (NifENB). Annotated Nif sequences were obtained from InterPro from 963 culture-derived genomes. Nif sequences were aligned individually and concatenated to form one NifHDKENB sequence. Phylogenies obtained using PhyML, FastTree, RapidNJ, and ASTRAL from individuals and concatenated protein sequences were compared and analyzed. All six genes were found across the Actinobacteria, Aquificae, Bacteroidetes, Chlorobi, Chloroflexi, Cyanobacteria, Deferribacteres, Firmicutes, Fusobacteria, Nitrospira, Proteobacteria, PVC group, and Spirochaetes, as well as the Euryarchaeota. The phylogenies of individual Nif proteins were very similar to the overall NifHDKENB phylogeny, indicating the assembly proteins have evolved together. Our higher resolution database upheld the three cluster phylogeny, but revealed undocumented horizontal gene transfers across phyla. Only 48% of the 325 genera containing all six *nif* genes are currently supported by biochemical evidence of diazotrophy. In addition, this work provides reference for any inter-phyla comparison of Nif sequences and a quality database of Nif proteins that can be used for identifying new Nif sequences.

## 1. Introduction

The nitrogen biogeochemical cycle requires reduction of atmospheric nitrogen to ammonia. About half of the 413 Tg reactive nitrogen introduced annually to the biosphere is derived through biological nitrogen fixation by the prokaryotic nitrogenase enzyme complex, with 140 Tg.yr^−1^ fixation in marine environments and 58 Tg.yr^−1^ through non-agricultural terrestrial fixation, and only 2% (5 Tg.yr^−1^) contributed by lightning [1]. The other half is contributed through a combination of synthetically fixed nitrogen and agricultural promotion of bacterial fixation in legumes. Biological nitrogen fixation or diazotrophy probably evolved 3.6–3.2 Ga ago to support expansion of biota in the nitrogen poor environment of that era [2,3]. Today it is the second most vital process for life on earth after photosynthesis [4]. Diazotrophs are divided into two major types: (1) symbiotic nitrogen-fixing bacteria which form symbiotic relation with legumes like *Rhizobium*, with actinorhizal plants such as *Frankia*, and *Cyanobacteria* associated with cycads, and (2) free-living nitrogen fixers belonging to genera such as *Azotobacter* and *Clostridium* [5].

Irrespective of their lifestyle all diazotrophs have a nitrogenase multi-subunit enzyme complex which is the only known natural system that catalyzes the breakdown of the triple bond between two nitrogen atoms in N_2_ [6]. This oxygen-sensitive nitrogenase complex has evolved into three variants; molybdenum nitrogenase (Nif), the rarer vanadium nitrogenase (Vnf), and iron-only nitrogenase (Anf) [7]. Vnf and Anf nitrogenase are also known as alternative nitrogenase. An oxygen insensitive nitrogenase was reported in *Streptomyces thermoautotrophicus* [8], but this has recently been refuted [9]. Molybdenum nitrogenase encoded by the *nifH*, *nifD*, and *nifK* genes are the most prevalent nitrogenase. Nitrogenase and its homologs have been classified into five phylogroups based on NifH and NifD proteins and their homologs by Raymond et al. [10]. Group I contains Mo-dependent nitrogenase from aerobic and facultative bacteria and II contains the Mo-dependent nitrogenase from anaerobic bacteria. Group III contains both the alternative forms of nitrogenase. Groups IV and V contain uncharacterized nitrogenase homologs and chlorophyll/bacterio-chlorophyll biosynthesis genes, respectively. Even though Nif homologs in Groups IV and V were thought to be unable to reduce nitrogen, one report suggested that *Endomicrobium proavitum*, which encodes a type IV nitrogenase, can fix nitrogen [11].

NifH is the most sequenced and studied of the three core nitrogenase components, NifH, NifD, and NifK [12]. Since development of the first *nifH* PCR primers by Zehr and McReynolds in 1989, the number of complete *nifH* sequences has skyrocketed from 1500 [13] to more than 8000 sequences at present, available in a curated NifH database (https://wwwzehr.pmc.ucsc.edu/nifH_Database_Public/, accessed on 2 October 2020). NifH phylogeny conducted by Zehr et al. in 2003 is one of the most comprehensive and is in close agreement with other NifH phylogenies [14,15]. They classified NifH into four clusters. Cluster I consists of Mo-containing *nifH* and some *vnfH*, Cluster II consists of *anfH* and some *nifH* from Archaea, and Cluster III consists of *nifH* sequences from a diverse group of anaerobic bacteria. Cluster IV consists of *nifH* homologs, some of which are uncharacterized *nif*-like sequences and others are chlorophyllide reductase genes. According to a cross-system comparison of *nifH* diversity using 16,989 publicly available *nifH* sequences, Cyanobacteria and the Proteobacteria are the most common taxa containing a *nifH* gene [16]. These authors also reported that soil has the most diverse *nifH* sequences compared to marine or mat habitats.

**Figure 1:**
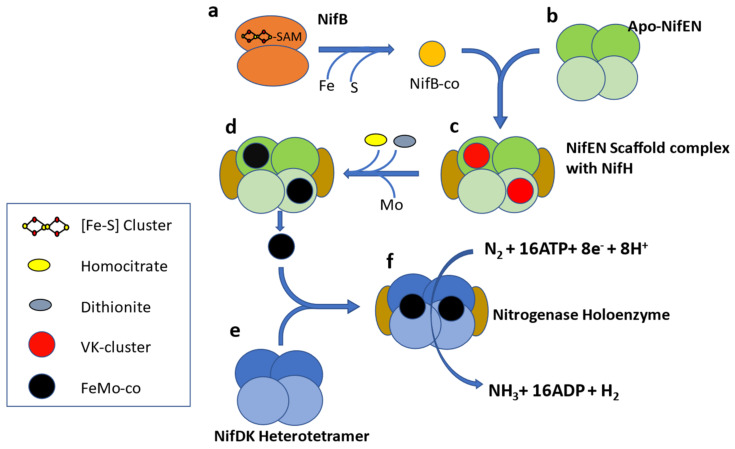
Schematic representation of FeMo-co biosynthesis. NifB (**a**) is a homodimer and belongs to the SAM radical protein family. NifB supplies homocitrate and Mo free, Fe-S cluster (precursor of Fe-Mo cofactor) to the Apo-NifEN heterotetramer (**b**). NifEN is homologous to NifDK and acts as molecular scaffold for assembly of Fe-Mo cofactor along with NifH (**c**,**d**). The assembled Fe-Mo cofactor is transferred to the Nitrogenase apoenzyme (**e**), which forms the nitrogenase holoenzyme (**f**) along with NifH. NifH has a role in both assembly of the Fe-Mo cofactor as well as reduction of dinitrogen [27].

Molybdenum nitrogenase is found in all diazotrophs and is a complex enzyme with two components. Component I, or Dinitrogenase, is a heterotetramer of NifD and NifK proteins which contains an iron-molybdenum cofactor (FeMo-co) in the active site of NifD. The second component, or Dinitrogenase reductase, is a NifH homodimer [17] (Figure 1). In addition to these structural proteins, several ancillary proteins are required for diazotrophy, including but not limited to proteins for FeMo-co biosynthesis (NifENBXUSVYQ), nitrogenase maturation (NifZM), and *nif* gene expression activation (NifA) [18]. In some diazotrophs, NifA activity is controlled by the anti-activator NifL [19], so the list of accessory proteins varies according to the species and physiological condition under which it fixes nitrogen. A study of 2000 complete genomes available in 2012 led to proposing NifHDKENB (Figure 1) as the minimum criteria for computational prediction of diazotrophy [20]. This six gene criterion has been widely used as diagnostic for diazotrophy in culture-independent studies [21,22,23,24].

The most updated phylogenies of nitrogenase are from 2011 based on NifH only [16], and 2013 using NifHDK [25], and introduction of multiple *nifH* primers has caused a surge of *nifH* sequences which in turn has increased the tendency to assign diazotropy to new species without strong biochemical evidence. This has led to a significant deviation from the gold standard of biochemical evidence and caused potential incorrect assignment of phylogeny to new sequences. Hence there is an acute need for a rigorously curated *nif* database based on high quality sequences obtained from the complete genomes of cultured isolates. We exploited the recent surge in the number of species with fully sequenced genomes (28,483) to conduct a phylogenetic study of diazotrophs using the six core Nif proteins H, D, K, E, N, and B from publicly available complete genomes. Nif genes were assigned to taxa using a reference phylogeny of bacterial and archaeal genomes, based on a comprehensive set of 381 marker genes [26] to present a clear view of distribution of diazotrophic species among different prokaryotic phyla. This holistic approach also provides a curated database of six Nif proteins and identifies several genera hitherto not known to fix nitrogen but with potential to do so. The higher resolution of this phylogenetic analysis enabled us to track the evolution and horizontal gene transfer of nitrogenase in individual phyla.

## 2. Materials and Methods

Annotated NifH, NifD, NifK, NifE, NifN, NifB, VnfH, VnfD, VnfK, AnfH, AnfD, and AnfK protein sequences were downloaded from the InterPro database (www.ebi.ac.uk/interpro/ accessed on 4 April 2019). Since InterPro is an integrated database built upon the signatures from several member databases like Pfam, PANTHER, and TIGRFAMs, annotation in InterPro is more reliable than individual databases. Sequences were grouped together by taxon name and only genome sequences having all the six genes (*nif*HDKENB) were selected for further study. In cases where there is more than one copy of *nif* gene per organism, only that *nif* gene occurring in the same genomic neighborhood of the remaining minimal *nif* gene complement was manually selected to yield a single set of *nif*HDKENB sequences for each organism. Organisms with fused *nif*EN and *nif*NB were also identified based on their sequence length and included. The genomes of organisms containing the defined *nif* genes were obtained from NCBI (www.ncbi.nlm.nih.gov, accessed on 5 April 2020) using CDS IDs obtained from mapping function in uniport (www.uniprot.org, accessed on 5 April 2020). Protein sequences are included as Supplemental Data S1.

Biosample IDs associated with the genomes in NCBI were used to obtain the attributes describing sample type (cultured or metagenome), source of isolation, assembly status, biotic relationship (free-living or host associated), host (if applicable), env_material, and env_feature. These attributes were used to get information on the habitat and lifestyle adopted by diazotrophs. All pertinent information is included as Supplemental Data S1 and S2.

Protein sequences were aligned individually using ClustalW multiple sequence alignment (version 2.1) in Galaxy (https://usegalaxy.org/, accessed on 7 August 2019) with default parameters and concatenated in R using the Phylotools package (version 0.2.2). Phylogenetic trees from the concatenated sequences were constructed using PhyML 3 [28], FastTree V2.1.10 [29] using the JTT+CAT evolution model, and RapidNJ [30] using the Kimura model. For both FastTree and RapidNJ default amino acid substitution model was used in the final analysis as other models also produced similar tree topologies. Branch support for PhyML 3 was calculated using a Bayesian-like transformation of aLRT (aBayes) [31] as it was the only method computationally feasible to run at HPC facility. Phylogeny of NifHDKENB was also compared by combining individual Nif trees as well. ASTRAL-III [32] was used to obtain a combined tree from six individual Nif trees obtained by using FastTree using the JTT+CAT evolutionary model. The ASTRAL tree was compared with concatenated trees by forming a consensus tree of three concatenated trees by using consensus clustering of phylogenetic trees obtained using Rphylip [33] which is an R implementation of Phylip [34]. Trees were annotated using iTOL [35] and Fig Tree v1.4 [36].

The 16S rRNA sequences were obtained from genomic assemblies in NCBI using the annotation key words 16S rRNA or 16S ribosomal RNA. The tree of life proposed by Zhu et al. [26] was used to delineate distribution of diazotrophs across taxa and to estimate geological time for the evolution of diazotrophy in different phyla.

## 3. Results

**Figure 2:**
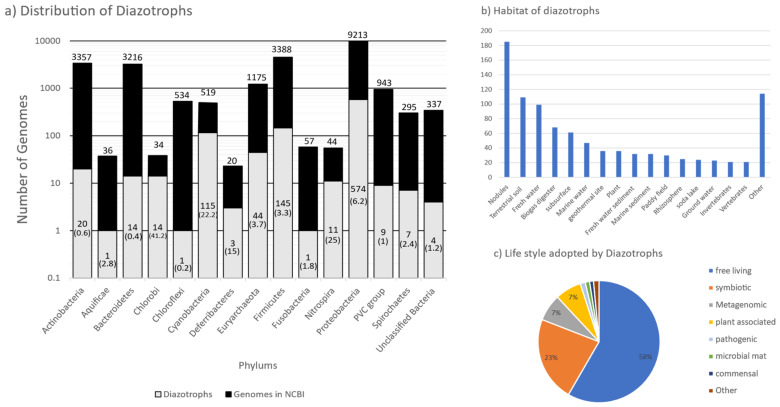
Distribution, habitat, and lifestyle of diazotrophs in various bacterial and Archaeal phyla. (**a**) The black bar represents the total number of genomes sequenced, and the gray bar the total number of diazotrophs in that phylum. Labels on top of black bar indicate the total number of sequenced genomes. Labels in the gray bar represent the number of genomes containing NifHDKENB in that phylum (numbers in parentheses are the percentage of sequenced genomes that have NifHDKENB). (**b**) Distribution of organisms having all six nif genes by habitat and (**c**) pie diagram showing the lifestyle adopted by various diazotrophs.

**Figure 3:**
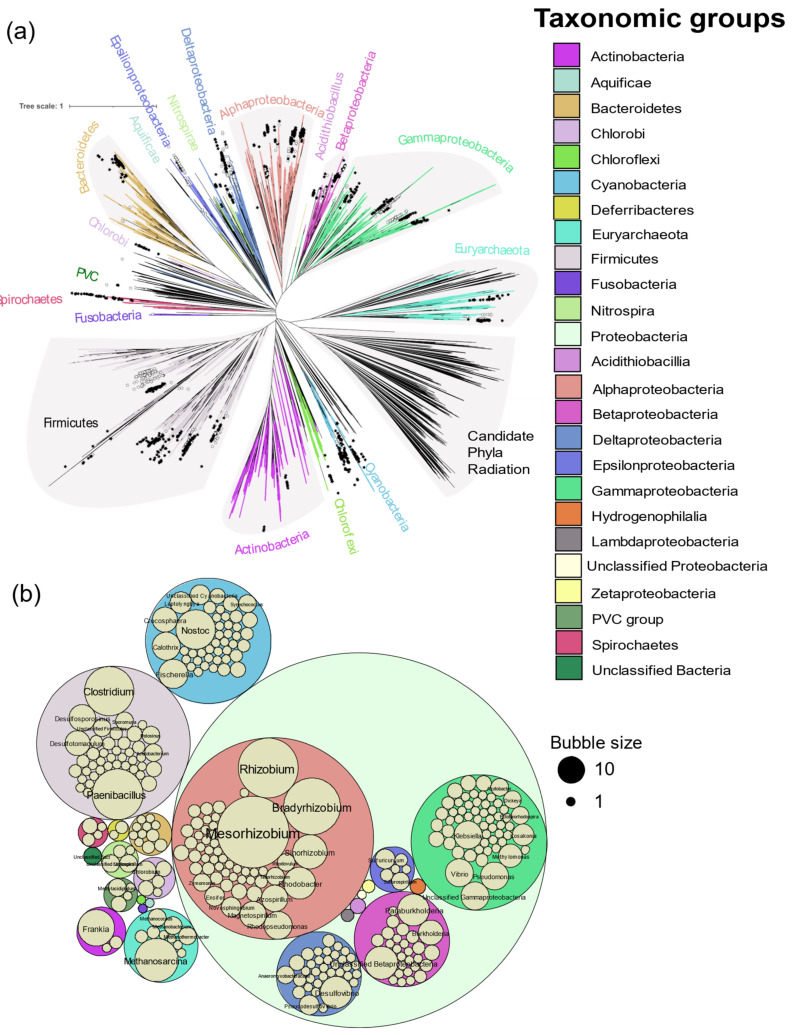
Distribution of diazotrophs by genus. (**a**) Distribution of diazotrophs in the tree of life proposed by Zhu et al. [23]. Nexus tree file for all prokaryotes was visualized in iTOL and leaves were annotated by genus according to the evidence of diazotrophy observed in this study. Solid and open symbols represent genera with and without biochemical evidence respectively. Genera without a star had neither genomic nor biochemical evidence of diazotrophy. (**b**) Distribution of the diazotrophic genera by the number of species observed in each genus. Bubble size corresponds to the number of assemblies of that genus and phylum.

In total, 963 genomes or genomes from metagenomes containing all six *nif* genes were obtained, falling into 325 genera from Actinobacteria, Aquificae, Bacteroidetes, Chlorobi, Chloroflexi, Cyanobacteria, Deferribacteres, Euryarchaeota, Firmicutes, Fusobacteria, Nitrospira, Proteobacteria, PVC group, and Spirochaetes (Figure 2). Of the 24,168 genomes available in NCBI (5 April 2020), *nif* genes were found in limited proportion, 0.2 to 41.1% per phylum, showing the wide but patchy distribution of diazotropy in both bacterial and archaeal phyla (Figure 2 and Figure 3). Most organisms were from Proteobacteria, followed by Firmicutes and Cyanobacteria. Some smaller phyla like Aquificae, Chloroflexi, and Fusobacteria had only one representative each. *Mesorhizobium* with 66 strains was the most sequenced diazotroph followed by *Rhizobium* (44), and *Bradyrhizobium* (41) (Figure 3).

Most of the genomes (867) were from cultured isolates, followed by metagenomic assembly (93), and single cell genomes (3). Diazotrophic organisms were from a wide range of habitats (Figure 2). The largest proportion of diazotrophs were isolated from root nodules (180), followed by terrestrial soil (109), fresh water (99), and biogas digesters (68). More than half (562) of the organisms were free living followed by a symbiotic lifestyle. Most of the symbiotic bacteria were rhizobia (165), fixing nitrogen in root nodules of leguminous plants. Other modes of symbiosis observed were root nodules in actinorhizal plants by *Frankia* sp. (15), cyanobacterial endosymbionts of diatoms (3), syntrophic coculture (*Syntrophobotulus glycolicus*, *Chlorobium ferrooxidans*), phototrophic consortium (*Chlorobium chlorochromatii*), Proteobacteria from gill tissue of bivalves (5), Spirochaetes from termite gut (3), *Nostoc* in coralloid roots (3), moss carpet (5), and Azolla (1). Thirty-six strains were isolated from thermophilic sites and eight strains isolated from psychrophilic sites (Supplementary Data S2).

### 3.1. Biochemical Evidence of Diazotrophy

Incorporation of ^15^N_2_ and the acetylene reduction assay have been used to biochemically confirm diazotrophy. With the growing ease of genome sequencing, isolates can be tentatively reported to fix nitrogen based on occurrence of a *nif* operon in their genome. Of the 325 genera containing *nif*HDKENB genes, 156 were without biochemical evidence of diazotrophy and most of them were represented by single strains (Supplemental Data S3). Figure 3 shows the distribution of prokaryotic genera with and without biochemical evidence in a tree of life proposed by Zhu et al. [26]. No biochemical evidence was found in the phyla Aquificae and Fusobacteria, both of which are represented here by a single strain. Most of the isolates in the delta and epsilon subdivisions of the Proteobacteria do not have biochemical evidence of N_2_ fixation to date.

### 3.2. Phylogeny of Nitrogenase

A total of 6 genes were obtained from all 963 organisms, but 29 assemblies from Cyanobacteria and Nitrospira had fused *nifE* and *nifN*, and 44 assemblies from Clostridia which had fused *nifN* and *nifB*. In addition to the Mo-Fe nitrogenase, 31 assemblies had vanadium containing V-Fe nitrogenase and 44 assemblies had iron only containing Fe-Fe nitrogenase. Species of *Azotobacter*, *Clostridium*, *Methanosarcina*, *Paenibacillus*, and *Rhodopseudomonas* were found to harbor all three forms of nitrogenase. Not all alternative nitrogenases were found to carry their own cofactor synthesis genes (*nif*ENB), hence phylogenetic analysis with six genes was limited to the Mo-Fe nitrogenase only. Phylogenetic analysis of Mo-Fe nitrogenase was done using multiple approaches. Reconstruction of species trees from six individual Nif protein trees (Supplementary Figures S1–S6) using ASTRAL-III gave similar patterns of clustering to the tree constructed using concatenated sequences. Trees constructed with multiple algorithms (PhyML, FASTTREE, and Rapid NJ) from concatenated sequences also agree on the major clusters (Supplementary Figures S7–S9).

**Figure 4:**
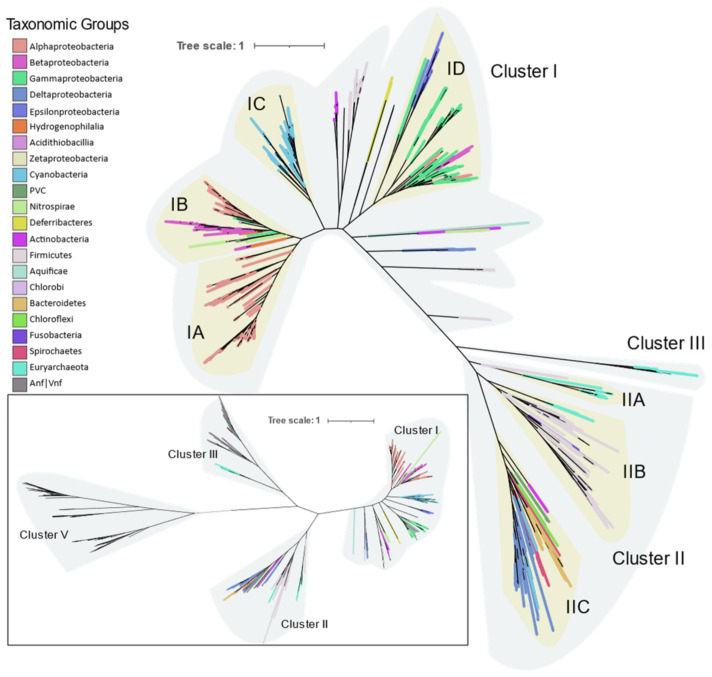
Molecular phylogenetic analysis of concatenated NifHDKENB proteins by FastTree using the JTT+CAT evolution model. Each clade is highlighted by the bacterial or archaeal phylum and Proteobacteria are further divided into classes. Inset: Phylogenetic analysis of the NifHDK protein sequences with concatenated bclLNB as outgroup. All the clusters are labeled as suggested by Raymond [10].

Phylogeny of the Mo-Fe nitrogenase using concatenated NifHDKENB obtained by FASTTREE is given in Figure 4 and the clusters are labeled according to Raymond et al. [10]. Cluster I contains aerobic and facultative bacteria from Proteobacteria, Cyanobacteria, Firmicutes, Actinobacteria, and other smaller phyla Aquificae, Deferribacteres, and Nitrospira. Cluster II consists of anaerobic bacteria (Firmicutes, Bacteroidetes, Chlorobi, Chloroflexi, Fusobacteria) and Archaea (Euryarchaeota). Cluster III consists of Mo-Fe nitrogenase from the Methanomada clade of Euryarchaeota and alternative nitrogenase as evident from the NifHDK tree (inset in Figure 4). Cluster III in the *nif*HDKENB tree appears small compared to *nif*HDK because alternative nitrogenases were not included as cofactor biosynthesis proteins were not universally present in alternative nitrogenase operons.

### 3.3. Distribution and Phylogeny of Nitrogenase in Various Phyla

#### 3.3.1. Proteobacteria

Proteobacteria is the largest bacterial phylum and consists of bacteria with diverse morphology and physiology, yet are united by 16S rRNA phylogeny. It had the largest number of representatives in this study as well (574). The nitrogenases of Proteobacteria occur entirely in Cluster I, with the exception of the Deltaproteobacteria whose nitrogenases cluster together with other anaerobic bacteria in Cluster II.

**Figure 5:**
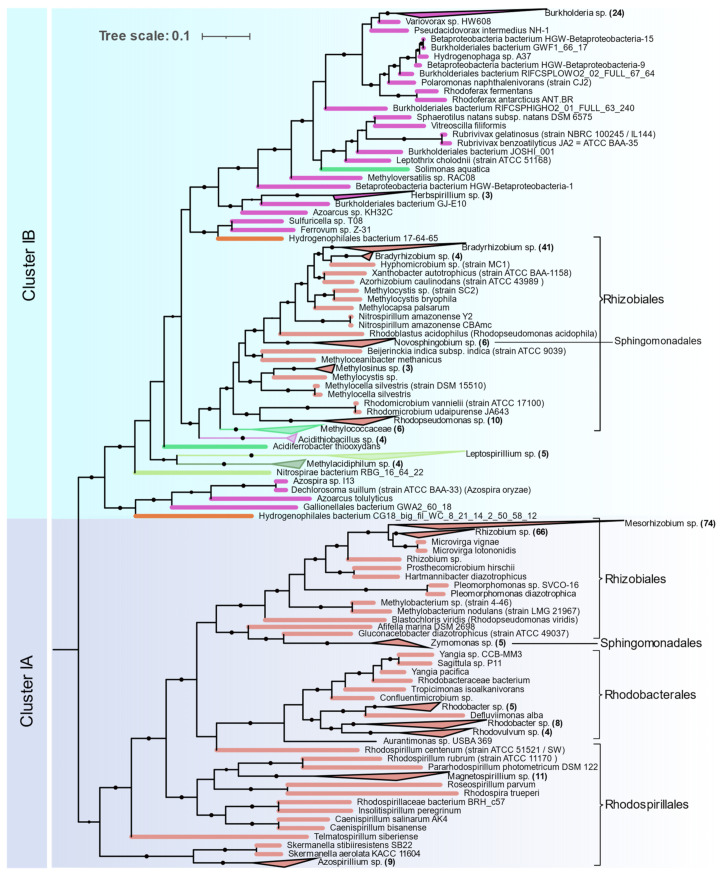
NifHDKENB Clusters IA and IB showing the phylogenetic relationship among Proteobacterias obtained by FastTree.

**Alphaproteobacteria** are distributed in Clusters IA and IB (Figure 5). The orders Magnetococcales, Rhodospirillales, Rhodobacterales, Sphingomonadales, and Rhizobiales were found to contain nif genes. Cluster IA is coherent with 16S rRNA phylogeny of Alphaproteobacteria where primitive Rhodospirillales, Rhodobacterales, and Sphingomonadales branch out earlier than Rhizobiales. Rhizobiales are distributed between Cluster IA and IB. Phyllobacteriaceae, Rhizobiaceae, and Rhodobiaceae form a monophyletic cluster in Cluster IA whereas Bradyrhizobiaceae, Xanthobacteraceae, and Beijerinckiaceae form a monophyletic cluster in IB along with Betaproteobacteria and other acidophilic methanotrophic bacteria. Some Alphaproteobacteria (*Magnetococcus*, *Magnetovibrio*, *Martelella*, and *Cohaesibacter*) occur in Cluster ID along with Gammaproteobacteria.

Nitrogenases from **Betaproteobacteria** are present in Cluster IB and are represented by the orders Ferrovales, Neisseriales, Nitrosomonadales, and Burkholderiales. The Rhodocyclales are present in Cluster ID. The Burkholderiales were the most abundant diazotrophic Betaproteobacteria in this study.

**Gammaproteobacterial** nitrogenases form a phylogenetically coherent cluster in ID. Enterobacterales and Pseudomonadales were the most common diazotrophic Gammaproteobacterial. Methanotrophic Methylococcales form a deep branch in the Rhizobiales cluster in IB.

Nitrogenases from **Deltaproteobacteria** occur in a monophyletic group in Cluster IIC except for the orders *Desulfuromonadales* and *Myxococcales* which occur at the base of cluster I. Most of the *Deltaproteobacteria* genomes in Cluster IIC are from *Desulfovibrionales* and *Desulfobacterales* and for several of these isolates, no biochemical evidence could be found (Supplement File S3). Distribution of nitrogenase in Deltaproteobacteria in Cluster IIC is also coherent with 16S rRNA phylogeny. Similarly, *Desulfuromonadales* (strictly anaerobic) and *Myxococcales* (aerobic/facultative anaerobic) that occur in Cluster I were found to be phylogenetically distinct from the rest of the Deltaproteobacteria in a recent phylogenetic study [26].

**Epsilonproteobacteria** are the next most abundant Proteobacteria represented by 15 isolates, all of which occur in a monophyletic cluster in ID along with Gammaproteobacteria.

#### 3.3.2. Archaea

**Figure 6:**
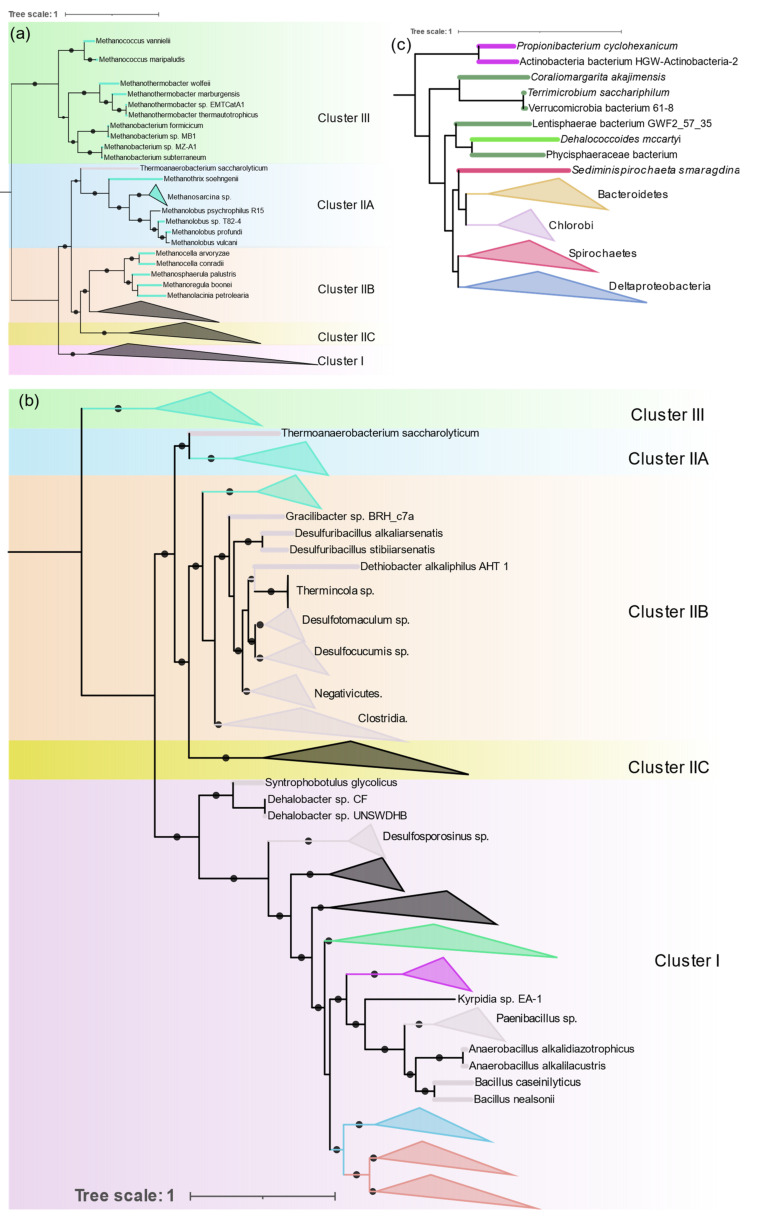
NifHDKENB phylogeny highlighting nitrogenase distribution in Archaea (**a**), Firmicutes (**b**), and overall distribution of Cluster IIC (**c**), obtained by FastTree, and manually rooted based on the position of the outgroup (Cluster V) in the NifHDK tree.

Diazotrophic Archaea were only found in methanogenic Euryarchaeota, but they are distributed in both Clusters II and III (Figure 6a). *Methanosarcina* is the most sequenced diazotrophic archaea (24 out of 44 genomes). Its nitrogenase forms a single monophyletic cluster in IIA along with other *Methanosarcinales* (*Methanothrix* and *Methanolobus*). *Methanomicrobiales* (*Methanosphaerula*, *Methanolacina*, and *Methanoregula*), and *Methanocellales* (*Methanocella*) form a monophyletic group in ClusterIIB together with *Firmicutes*. Nitrogenases from the group “Methanomada” (*Methanococcales* (*Methanococcus*), and *Methanobacteriales* (*Methanobacterium*, *Methanothermobacter*)) are different from any archaeal nitrogenase and form a deep branch in Cluster III along with the alternative nitrogenases. Nif phylogeny strictly follows the 16S rRNA phylogeny, indicating the vertical transfer of *nif* genes within Archaea.

#### 3.3.3. Firmicutes

Genomes of Firmicutes (145) from Bacilli (41), Clostridia (90), Negativicutes (13), and Tissierellia (1) were found to contain all six *nif* genes. *Clostridium* and *Paenibacillus* are the most prevalent diazotrophs in Firmicutes. *Nif* phylogeny shows three distinct evolutionary patterns of nitrogenase in Firmicutes (Figure 6b). Although distribution of Firmicute *nif* appears to be patchy and interspersed by other taxa, within each patch 16S rRNA phylogeny is highly conserved. All Bacilli (except *Desulfuribacillus*) are present as a monophyletic cluster in Cluster I together with aerobic high G + C gram positive Actinobacteria. Paenibacillaceae (*Paenibacillus* and *Fontibacillus*), and Bacillaceae (*Anaerobacillus* and *Bacillus*) form their own distinct cluster and *Kyrpidia* branches out early, which is in close harmony with the 16S rRNA phylogeny of the Bacilli. Only two *Bacillus* (*B. nealsonii* and *B. caseinilyticus*) were found to contain complete *nif* operons.

In Cluster IIB Firmicutes occur as a monophyletic cluster which comprises Clostridia, Negativicutes, and Tissierellia. Peptococcaceae (*Desulfosporosinus*, *Dehalobacter*, *Syntrophobotulus*, and *Desulfitobacterium*) occur at the base of Cluster I. The division of Peptococcaceae into two phylogroups is also evident in 16S rRNA phylogeny of Peptococcaceae [37].

*Thermoanaerobacterium* which branches deep in 16S rRNA phylogeny also has very distinct *nif* genes that branch out earlier than other Firmicutes and occur in IIA.

#### 3.3.4. Cyanobacteria

**Figure 7:**
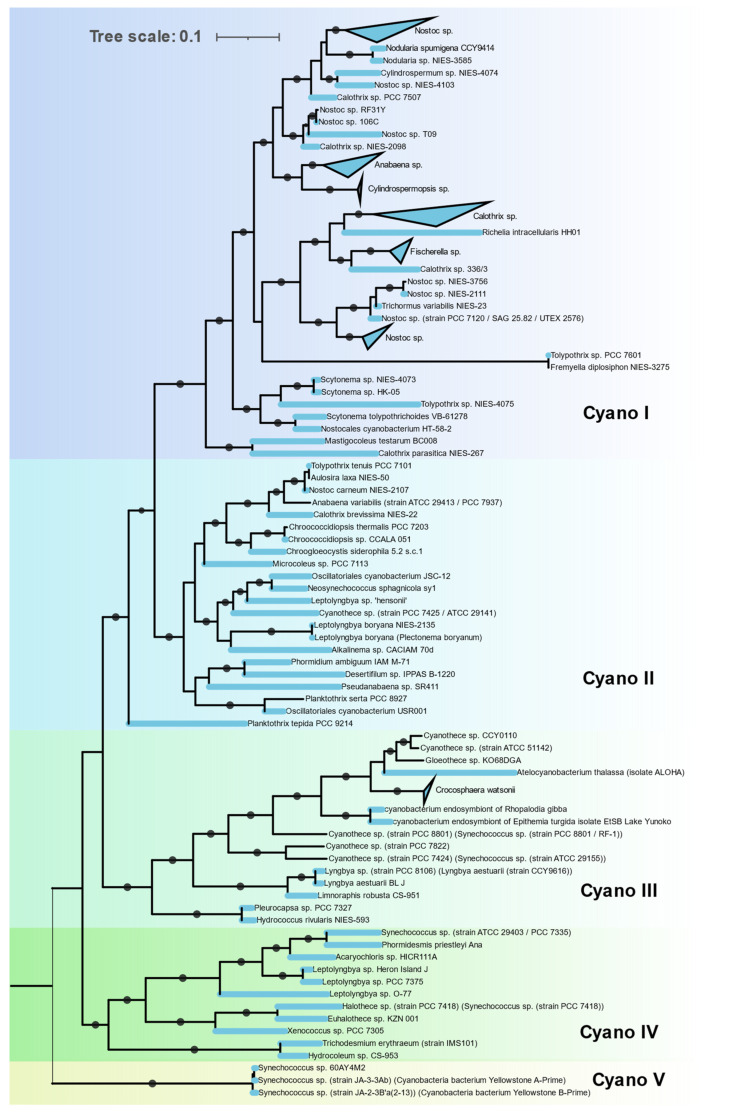
Phylogenetic relationship among Cyanobacteria in Cluster IC of the Concatenated NifHDKENB tree obtained by FastTree.

The Nif proteins of all 115 cyanobacterial genomes clustered together in a single cluster (Cluster IC) with no representatives of non-cyanobacterial taxa (Figure 7). This indicates a monophyletic origin of cyanobacterial *nif* genes. The *nif* phylogeny agrees with the 16S rRNA phylogeny of these cyanobacterial isolates, indicating all the cyanobacteria derived their *nif* operon from a common cyanobacterial ancestor. Cyanobacteria that possess *nif* genes were found to occur in various habitats from hot springs (*Synechococus* sp. Strain JA-2-3B’a-2-13), antartic endolith (*Chroococcidopsis* sp.), marine (*Trichodesmium erythraeum*, *Crocosphaera*) to fresh water (Oscillatoria). Within the cyanobacterial cluster these isolates form five distinct clades.

Clade I is homogenous in terms of 16S rRNA phylogeny, containing all the Nostocales. These are filamentous cyanobacteria producing specialized, terminally differentiated cells called heterocysts for nitrogen fixation. Since photosynthesis and nitrogen fixation are spatially separated from each other, these cyanobacteria are capable of fixing nitrogen in aerobic condition [38]. Major representatives in this clade are *Nostoc*, *Fischerella*, *Calothrix*, *Nodularia*, and *Anabaena*.

Clade II is polyphyletic, containing Synechococcales, Oscillatoriales, Chroococcidiopsidales, and a single representative of Chroococcales, *Chroogloeocystis* where no biochemical evidence has been reported. Chroococcidiopsidales are unicellular, free-living cyanobacteria shown to fix nitrogen in anaerobic condition only [39]. Other cyanobacteria in this clade are all filamentous but lack heterocysts and fix nitrogen in anaerobic or microaerophilic conditions only [40].

Clade III consists of unicellular Pleurocapsales and Chroococcales along with single filamentous genus *Lyngbya*. All Chroococcales of Cluster III form a single cluster and contain some of the most important marine diazotrophs (*Crocosphaera*/UCYN B, *Atelocyanobacterium*/UCYN A, *Gloethece*, *Rippkaea*, and *Zehria*). Chroococcales use temporal separation of photosynthesis and diazotrophy by fixing nitrogen in the dark cycle only [41].

Clade IV is also polyphyletic, consisting of Synechococcales, Pleurocapsales, Oscillatoriales, and Chroococcales.

Clade V contains three unique cyanobacteria characterized by deep branching from the rest of the cyanobacteria in both 16S rRNA and NifHDKENB. These are unicellular, thermophilic cyanobacteria isolated from hot springs in Yellowstone National Park [42]. Although all three strains have all six *nif* genes, diazotrophic growth has not been reported yet.

#### 3.3.5. Actinobacteria

There were 21 Actinobacterial genomes containing all 6 *nif* genes, 18 were *Frankia*, 1 *Propionibacterium*, and 2 unclassified actinobacteria. All the *Frankia* make a monophyletic cluster in Cluster I (Figure 6b) along with other gram-positive bacteria. Nevertheless, anaerobic *Propionibacterium* and one of the unclassified actinobacteria cluster together with *nif* from *Verrucomicrobium* in Cluster IIC (Figure 6). The remaining unclassified actinobacterial *nif* align *nif* from Nitrospira. All *nif* containing Actinobacteria cluster together by 16S rRNA indicating polyphyletic evolution of nitrogenase in Actinobacteria.

#### 3.3.6. Bacteroidetes and Chlorobi

These phylogenetically related phyla occurred as a monophyletic group in Cluster IIC (Figure 6c) with a separate branch for each phylum. Only 11 genera (*Bacteroides*, *Dysgonomonas*, *Paludibacter*, *Azobacteroides*, *Labilibaculum*, *Alkalitalea*, *Geofilum*, *Saccharicrinis*, *Draconibacterium*, *Lutibacter*, and *Arcticibacter*) in Bacteroidetes were found to have all *nif* genes, of which only five have been shown to fix nitrogen. Chlorobi is a small phylum of green sulfur bacteria and all the sequenced members of this phylum have *nif* genes except *Ignavibacteria*. The evolution of nitrogenase in these two phyla is in close alignment with their 16S RNA phylogeny.

#### 3.3.7. Spirochaetes

All the Spirochaete nitrogenases form a monophyletic cluster in IIB (Figure 6c). All the diazotrophic Spirochaetes belonged to three genera (*Spirochaeta thermophila*, *Treponema azotonutricum*, *Treponema primitia*, and *Sediminispirochaeta smaragdinae*). All of them are strictly anaerobic.

#### 3.3.8. Other Anaerobes

Verrucomicrobia, Chloroflexi, Planctomycetes, and Lentisphaerae form a single cluster in IIB (Figure 6c). Verrucomicrobia, Planctomycetes, and Lentisphaerae are phylogenetically related and group together as the PCV clade. One methanotrophic genus of Verrucomicrobia, Methylacidiphilum forms a separate group in Cluster IB with Nitrospira and other acidophilic Gammaproteobacteria. The Planctomycetes and Lentisphaerae representative were obtained from metagenomic sequencing. The only Chloroflexi found to harbor nitrogenase was Dehalococcoides mccartyi (D. ethenogenes) and the Fusobacteria were also represented by a single observation, Ilyobacter polytropus, both of which occur in Cluster IIB.

#### 3.3.9. Nitrospira, Deferribacterales, and Aquificacea

These are small phyla represented by 10, 3, and 1 strain, respectively. Nitrospira and Aquificae form a single cluster at the base of Cluster I. Acidophilic *Leptospirillium* sp. of the Nitrospira occur at the base of Cluster IB. Deferribacterales form a single deep branch in Cluster ID.

### 3.4. Alternative Nitrogenases

**Figure 8:**
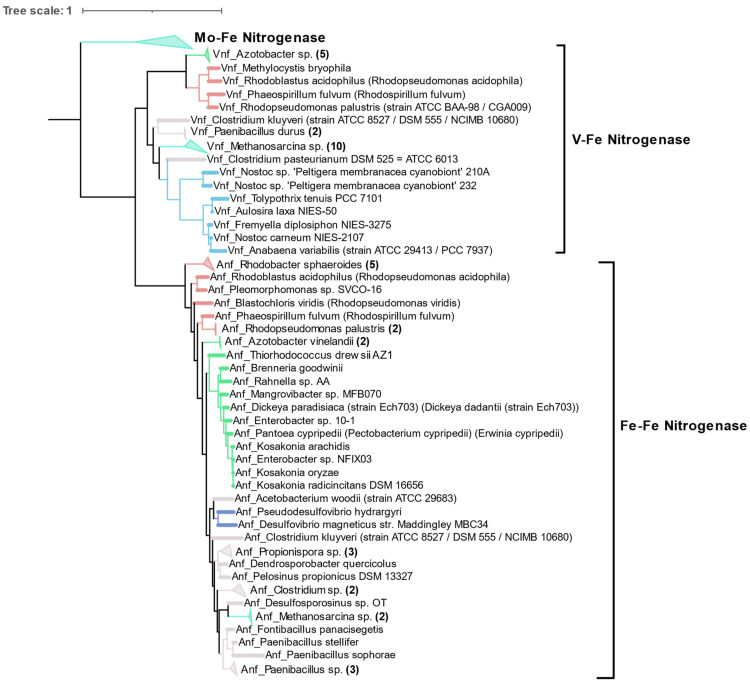
Cluster III of the concatenated AnfHDK/VnfHDK tree obtained by FASTTREE, showing the phylogenetic relationship between isolates carrying alternative forms of nitrogenase.

Compared to the diversity of Mo-Fe nitrogenases, alternative forms of nitrogenase are limited to very few taxonomic groups as shown by phylogeny of concatenated HDK proteins. Vanadium containing nitrogenase (Vnf) is found only in some Alphaproteobacteria, Gammaproteobacteria, Firmicutes, Archaea (*Methanosarcina* sp.), and Cyanobacteria. Similarly, Fe only containing nitrogenase (Anf) is found in some Alphaproteobacteria, Gammaproteobacteria, Deltaproteobacteria, Archaea (*Methanosarcina* sp.), and Firmicutes (Figure 8). Phylogenetically, Vnfs and Anfs form their own distinct clades, but are very similar to Mo-Fe nitrogenases from the Methanomada clade of Euryarchaeota. Anfs and Vnfs are very similar to each other, suggesting their very recent evolution from Mo-Fe nitrogenase (inset in Figure 4).

### 3.5. Horizontal Gene Transfer (HGT)

**Figure 9:**
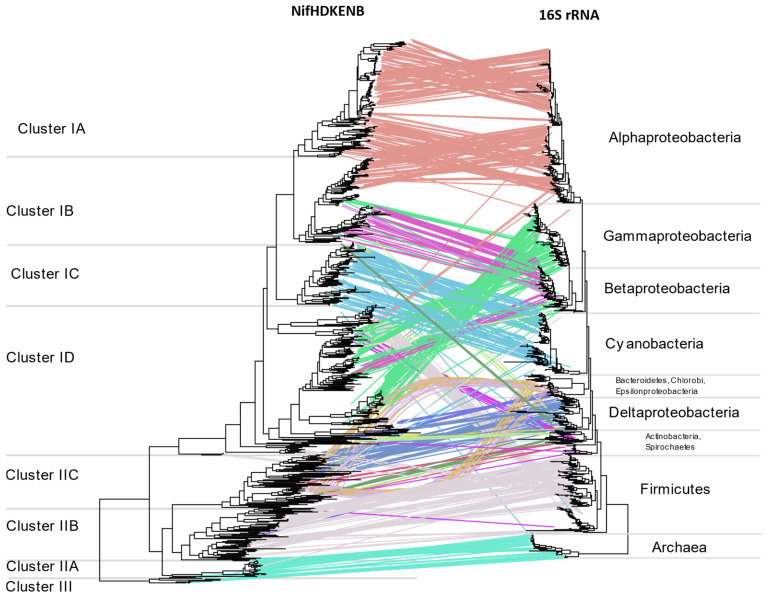
Tanglegram comparing the concatenated NifHDKENB tree with 16S rRNA phylogeny of the diazotrophs, both obtained using FastTree. Lines indicate the respective positions of the 963 bacteria in the 2 trees. See Supplementary Tree File for details of the horizontal gene transfer.

Multiple instances of HGT are evident when comparing *nif* phylogeny with16S rRNA phylogeny (Figure 9) or the tree of life reported by Zhu et al. [26] (Supplementary Figure S10). The two most evident instances of HGT are within anaerobic niches and acidic niches where methylotrophs are concentrated. Regardless of their 16S rRNA phylogeny, the NifHDKENB sequences of strict anaerobes belonging to diverse phyla were similar, aligning together in Cluster II. This included NifHDKENB sequences from strict anaerobes like Clostridia, Bacteroidetes, Chlorobi, PVC, Chloroflexi, Fusobacteria, Deltaproteobacteria, and Spirochaetes that cluster together in Cluster II with Euryarchaea. Within Cluster II, Clostridia and other anaerobes form two clear clusters, indicating two major instances of HGT. Details are apparent when zooming in to the Supplementary Tree File.

Cluster IB represents another instance of HGT where acidophilic and methanotrophic bacteria cluster together irrespective of their phylogeny. At the root of this cluster are *Methylacidiphilum* sp. from PVC, *Leptospirillium* sp. from Nitrospira, *Acidithiobacillus* from Proteobacteria, and species of Methylococcaceae from Gammaproteobacteria, all of which might have obtained their *nif* genes from ancient Alphaproteobacteria. In addition to these, there are also several instances where one or more genus appears to cluster with phylogenetically unrelated bacteria (Figure 9, Supplementary Tree File) indicating promiscuous lateral transfer during the early divergence of bacteria.

## 4. Discussion

Using the minimal structural (NifHDK) and assembly (NifENB) protein components of the nitrogenase enzyme as a marker of diazotrophy, we found diazotrophs in the Proteobacteria, Firmicutes, Cyanobacteria, Bacteroidetes, Chlorobi, Nitrospira, PVC group, Spirochaetes, Deferribacteres, Aquificae, Fusobacteria, Chloroflexi, and Euryarchaeota. In addition to these phyla, diazotrophy has been reported in Chrysiogenales [20], Acidobacteria [43] and Elusimicrobia [11], and *Azoarcus*-*Aromatoleum* groups [44] but these were not included in this study as at least one gene was absent or misannotated according to the KEGG database. Although the Candidate Phyla Radiation group (CPR) accounts for over 15% of bacterial diversity [45], no evidence of diazotrophy was found in the genomes available for this prokaryotic group. CPR bacteria have reduced genome size and lack basic pathways like the citric acid cycle and respiratory chains [46], indicating the primitive nature of these bacteria. Among non-CPR bacteria, Proteobacteria accounted for more than half of the diazotrophic species and some smaller phyla are represented by only a single species. While distribution of diazotrophs appears widespread across the non-CPR bacteria, only 3.9% of the bacterial whole genomes harbor the six core *nif* genes (Figure 2a). With the consumption of 16 ATPs per dinitrogen reduced, the nitrogenase system is very costly for a bacterium; hence it is energy efficient for species living in nitrogen-rich environments to rescind the nitrogenase enzyme. In fact, multiple studies have shown a decrease in diversity of diazotrophs when treated with nitrogen fertilizer [47,48]. Phyla represented by photosynthetic species like Cyanobacteria (22.2%) and Chlorobi (41.2%), that are primarily found in nitrogen deficient aquatic habitats, have significantly higher representation of diazotrophic members. This indicates that the maintenance of nitrogenase systems is heavily dependent on the scarcity of nitrogen in the niche, and coupling of the two most important biological pathways, photosynthesis and nitrogen fixation, is a more efficient method in fixing both C and N.

Traditionally, phylogeny of nitrogenase has been based on either *nifH* or *nifD* but use of one or both of these was reportedly prone to a false positive rate of diazotrophy [20]. By using all six genes, we found 84% of the sequenced isolates to have biochemical evidence, and the remaining 16% are recently isolated strains (after 2010) (Supplementary Figure S11). None of the information available for these strains included mention of experimental testing for diazotrophy, so these could all be new potential diazotrophs not yet characterized. Conversely, the literature abounds with reports of bacterial genera able to fix nitrogen but without genetic or biochemical evidence, for example the genus *Bacillus*. Most *Bacillus* found to have *nif* genes, such as *B. polymyxa* and *B. macerans*, have been shown to have multiple plant beneficial properties along with nitrogen fixation, and have been reclassified as *Paenibacillus* [49]. *B. nealsonii* and *B. caseinilyticus*, the only *Bacillus* that possesses all core six *nif* genes, are extremophiles and lack biochemical evidence for nitrogen fixation. This is in contrast to multiple claims of diazotrophic *Bacillus* isolates, supported only by culture evidence for growth on nitrogen free media [50,51,52,53]. However, none of these claims have been supported by genetic evidence. In addition to the archaeal diazotophs proposed by Leigh [54], potential diazotrophic species were observed in the orders *Methanocellales* and *Methanomicrobiales* as well. Although Actinobacteria is a large phylum where diazotrophy has been reported in multiple genera [55], only *Frankia* and *Propionibacterium* were found to have all six *nif* genes in this study.

The six gene criterion yielded nitrogenases aligning exclusively with Clusters I, II and III in the Raymond classification [10]. While several genomes contained *nifH* aligning with Clusters IV or V, none had all six genes, with at least one of the *NifENB* gene homologs either missing or not annotated in the KEGG database. As *nifH* of Clusters IV and V do not encode functional nitrogenases, this result strengthens the case for the use of six genes for probing potential diazotrophs. One notable exception is *Endomicrobium proavitum* reported by Zheng et al. [11] which was experimentally shown to fix nitrogen but harbors nitrogenase homologous to Group IV nitrogenase. However, *Endomicrobium* genomes available in the KEGG database lack *NifEN* genes. Many operons containing alternative nitrogenases *vnfHDK* or *anfHDK* did not have their own set of *ENB* genes. This is perhaps indicative that some bacteria encoding Group IV—NifHDK can express an active nitrogenase, even if they do not encode the NifEN assembly proteins. The exact mechanism for this is yet to be determined but suggests that there may be some exceptions to the six gene criteria established by Dos Santos et al. in 2012. Phylogenetically, the six *nif* genes have very similar distribution, suggesting that the *nif* operon evolved as a unit. Our analysis of the six genes points to the influence of habitat on the evolution of the nitrogenase enzyme. Smaller phyla with species adapted to a particular niche were found clustered together in a single cluster. Taxonomically unrelated species occurring in a particular niche were found to have similar *nif* genes, suggesting the role of lateral gene transfer in evolution of the nitrogenase enzyme. As an example, two distinct physiological groups of Firmicutes fell in two distinct NifHDKENB clusters. Anaerobic clostridia and related taxa aligned with archaea occurring in Cluster IIB, while the aerobic firmicutes occurred in Cluster I together with the Actinobacteria. On the other hand, Cyanobacteria, which comprise photosynthetic and mostly aquatic bacteria, occurred in a monophyletic clade which is in close agreement with its phylogeny supported by the study of Esteves-Ferreira et al. [56]. Species of larger phyla like Proteobacteria and Firmicutes which are adapted to a wide range of physiological conditions were found to have *nif* genes distributed across multiple clusters in the phylogenetic tree (Figure 4). Proteobacteria form phylogenetically coherent clusters except for *Bradyrhizobiaceae* and related families of Alphaproteobacteria (Figure 5), and Betaproteobacteria cluster together with acidophilic, methanotrophic bacteria from diverse taxonomical clades also observed by Khadem et al. [57] with methylotrophic Verrucomicrobium, *Methylacidiphilum*. Subcluster ID mainly consists of Gammaproteobacteria with some exceptions of Alphaproteobacteria like *Martellela*, *Cohesibacter*, *Magnetovibrio*, and Betaproteobacteria like *Aquaspirillium*, *Sideroxydans*, and some *Rhodocyclales*. Similarly, nitrogenase in Archaea is confined within the methanogenic Euryarchaeota. The Stenosarchaea group of Euryachaeota comprises most of the Archaeal diazotrophs, all of which occur in Cluster II together with other anaerobic bacteria. Smaller phyla like Bacteroidetes, Chlorobi, Spirochaetes, and the PVC group, which, in spite of the wide range of habitats, all clustered together in subcluster IIC, correlating with their anaerobic respiratory pathways.

**Figure 10:**
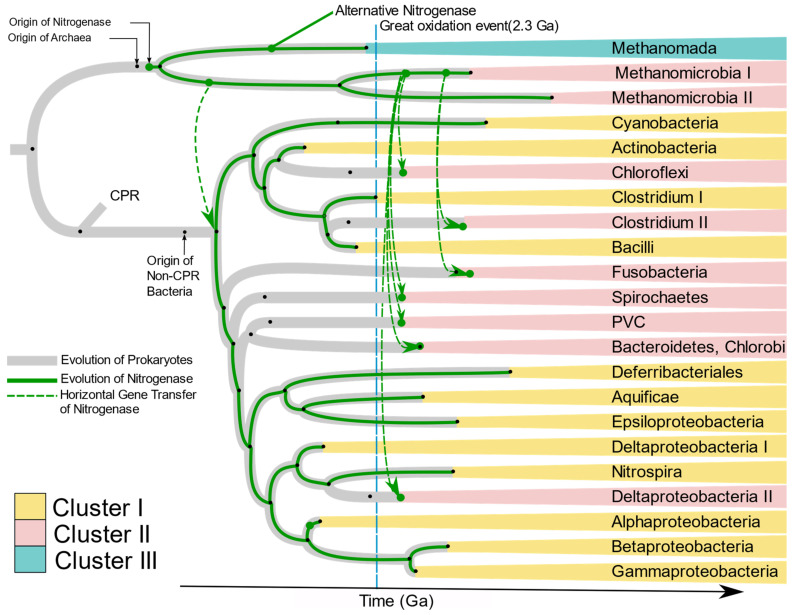
Proposed evolution of Diazotrophs. The gray tree represents the chronogram of prokaryotes while the green tree represents the proposed evolution of nitrogenase. The dashed green lines indicate horizontal gene transfer as supported by homology of NifHDKENB sequences. Black dots indicate origin of the respective phyla or groupings. The evolutionary timescale is based on Zhu et al., 2019 [26]. The Methanomada clade in Euryarchaeota includes orders *Methanobacteriales* and *Methanococcales*. Methanomicrobia I includes the orders *Methanocellales* and *Methanomicrobiales*. Methanomicrobia II includes *Methanosarcinales*. Clostridium I includes the family *Peptococcaceae*, Clostridium II includes Clostridia and Negativicutes. Deltaproteobacteria I includes *Myxococcales*, and Deltaproteobacteria II includes other Deltaproteobacteria.

Raymond et al. [10] proposed two plausible hypotheses of the evolution of nitrogenase. The first is that the nitrogen-fixing LUCA harbored nitrogenase, and present-day diversity was attained by loss of *nif* genes in large numbers of taxonomic groups including Eukaryotes and non-methanogenic archaea. The second hypothesis has nitrogenase originate in the methanogenic archaea, with transfer of *nif* genes to bacteria by HGT. Our results strongly support the latter hypothesis. The absence of *nif* genes in Candidate Phylum Radiation (CPR), Eukarya, and non-methanogenic Archaea, and branching of bacterial *nif* from archaea strongly suggests that nitrogenase originated first in primitive methanogenic Archaea. This hypothesis has been supported by Boyd and Peters [25]. The present phylogenetic analysis of *nifHDKENB* genes and the chronogram of prokaryotes proposed by Zhu et al. [26] (Supplementary Figure S10) enabled us to propose the evolution of nitrogenase in each phylum harboring diazotrophic species (Figure 10). Kasting and Walker [58] proposed that a nitrogen crisis occurred at ~3.5 Ga, overlapping with the origin of methanogenic Euryarchaeota (Supplementary Figure S10); hence nitrogenase must have evolved during that time in ancient methanogenic Euryarchaeota in response to the shortage of combined nitrogen. Ancient nitrogenase was vertically transferred to two groups of Euryarchaeota, Methanomada, and Stenosarchaea. An alternative nitrogenase originated from ancient Methanomada and forms Cluster III. The first *nif* in bacteria was laterally transferred to ancient bacteria from the ancient Methanomicrobia between 3.5 and 3.18 Ga, after which it was vertically transferred to all bacteria (Figure 10). The present scattered distribution of nitrogenase across multiple phyla likely originated through loss of nitrogenase by organisms adapted to live in nitrogen-rich environments, followed by multiple lateral transfers within anaerobic niches. The great oxidation event in 2.3 Ga. must have turned many bacterial environments aerobic, creating isolated anaerobic niches. This would have brought diverse anaerobes into close proximity in the available anaerobic niches. Co-occurrence of unrelated taxa in close proximity would increase exchange of genes, thus favoring HGT among anaerobes. This is supported by the similarity of *nif* genes of anaerobes like Bacteroidetes, Chlorobi, Spirochaetes, Deltaproteobacteria, Clostridium, and PVC to Methanomicrobia. Based on the branching pattern in Cluster II, two distinct HGT’s must have occurred to achieve the present distribution of *nif* genes among anaerobic bacteria (Figure 10), where Clostridium II and Fusobacteria obtained the genes much later than other anaerobes. Cluster I on the other hand is dominated by aerobic and facultative anaerobic diazotrophs, most of which belong to Alphaproteobacteria, Cyanobacteria, and Firmicutes. Although the majority of Alphaproteobacteria have lost the *nif* genes, they are preserved in the species which evolved to be in symbiosis with plants (*Rhizobiales*) and free-living species which primarily occur in nitrogen deficient habitats like water (*Rodospirillales* and *Rhodobacterales*). Occurrence of Aquificae and Nitrospirae at the root of Cluster I and clustering of Cyanobacteria, Actinobacteria, and aerobic firmicutes in the *nif* tree strongly suggests nitrogenase in these phyla must have evolved by vertical transfer from ancient non-CPR bacteria.

## 5. Conclusions

This study shows that the structural genes of nitrogenase (*nifHDK*) and Mo-Fe cofactor assembly proteins (*nifENB*) have very similar phylogeny, indicating that all six genes must have co-evolved. Additionally, the similarity of NifE with NifD, and NifK with NifN strongly supports the origin of NifEN by duplication of an ancestor NifDK. Using these six genes as criteria for the study of diazotophs, only potentially active diazotrophs were selected. All nitrogenases selected using this criterion could be classified into one of the Raymond Clusters, I, II, or III. Using all six genes also enabled us to understand the diversity of nitrogenase in individual phyla and the relationship between the nitrogenases in different phyla. Lateral gene transfer has played an important role in distribution of nitrogenase, hence multiple taxa in the same phylum can have very distinct nitrogenases based on their physiological and ecologically driven co-occurrence with unrelated taxa. Therefore, this work provides reference for any inter-phyla comparison of *nif* sequences and a quality database of Nif proteins that can be used for identifying new *nif* sequences and designing *nif* primers.

## Data Availability

Supporting data is available in Supplemental Data S1–S3 and the Supplemental Tree File.

## Supplementary Materials

The following are available online at https://www.mdpi.com/article/10.3390/microorganisms9081662/s1, Figure S1: Phylogenetic analysis of individual NifH proteins by FastTree using the JTT+CAT evolution model, Figure S2: Phylogenetic analysis of individual NifD proteins by FastTree using the JTT+CAT evolution model, Figure S3: Phylogenetic analysis of individual NifK proteins by FastTree using the JTT+CAT evolution model, Figure S4: Phylogenetic analysis of individual NifE proteins by FastTree using the JTT+CAT evolution model, Figure S5: Phylogenetic analysis of individual NifN proteins by FastTree using the JTT+CAT evolution model, Figure S6: Phylogenetic analysis of individual NifN proteins by FastTree using the JTT+CAT evolution model, Figure S7: Molecular phylogenetic analysis of concatenated NifHDKENB proteins by Maximum Likelihood (PhyML) with branch support by posterior probability. Each clade is highlighted by the bacterial or archaeal phylum and Proteobacteria are further divided into classes, Figure S8: Molecular phylogenetic analysis of concatenated NifHDKENB proteins Neighbour joining by RapidNJ using Kimura model. Each clade is highlighted by the bacterial or archaeal phylum and Proteobacteria are further divided into classes, Figure S9: Cladogram showing comparison of Astral tree obtained from six individual trees vs. majority consensus tree obtained from three trees obtained by FASTTREE, PhyML, and Neighbour Joining using concatenated NifHDKENB proteins. Black dots in Astral tree represents the Astral bootstrapping and on the concatenated tree represents the branching present in all three trees and all other splits present in at least two trees, Figure S10: Chronogram of evolution of diazotrophs obtained by selecting diazotrophic genera from the microbial evolution tree proposed by Zhu et al., 2019 using source data file. Node labels represent the time in billion years ago (Ga) in the original tree, Figure S11: Number of genomes containing all six *nifHDKENB* genes according to the year they were reported. Number of genomes without biochemical evidence increased rapidly with the increase in the number of genomes reported, Supplemental Data S1: Nif/Vnf/Anf HDKENB protein sequences used for analyses, Supplemental Data S2: Information on the strains and gene/protein sequences used, Supplemental Data S3: List of all genera for which biochemical evidence of nitrogen fixation could be found in the literature, Supplemental tree file: High resolution tanglegram comparing the concatenated NifHDKENB tree with 16S rRNA phylogeny of the diazotrophs, both obtained using FastTree. Lines indicate the respective positions of the 963 bacteria in the 2 trees.
