# Supplementary material for: Phylogeny of Nitrogenase Structural and Assembly Components Reveals New Insights into the Origin and Distribution of Nitrogen Fixation across Bacteria and Archaea
Source: Microorganisms. 2021 Aug 4;9(8):1662. doi: 10.3390/microorganisms9081662 (PMC8399215; doi:10.3390/microorganisms9081662)
Supplement: Supplementary file 1 [file microorganisms-09-01662-s001.zip › Archive 2/Supplement Data S3 Biochemical evidence.docx]

| **Genus** | **Frequency** | **Biochemical Evidence** | **Reference** | **Comments** | **First Genome Reported on** |
| --- | --- | --- | --- | --- | --- |
| Acetobacterium | 4 | No | NA | NA | 2012-02-13 |
| Acidiferrobacter | 1 | No | NA | NA | 2016-08-14 |
| Acidihalobacter | 1 | Yes | (Rosenberg et al., 2014) | NA | 2016-06-21 |
| Acidithiobacillus | 3 | Yes | (Dos Santos et al., 2012) | NA | 2008-12-18 |
| Afifella | 1 | Yes | (Madigan, 1995) | Previously *Rhodopseudomonas* | 2016-10-16 |
| Agarivorans | 1 | No | NA | NA | 2013-06-11 |
| Alkalibacter | 1 | No | NA | NA | 2016-12-02 |
| Alkalinema | 1 | Metagenome | NA | NA | 2017-05-17 |
| Alkaliphilus | 1 | No | NA | NA | 2007-06-27 |
| Alkalitalea | 1 | No | NA | NA | 2017-03-07 |
| Allochromatium | 2 | Yes | (Dos Santos et al., 2012) | NA | 2010-02-15 |
| Amphritea | 1 | No | NA | NA | 2016-10-30 |
| Anabaena | 2 | Yes | (Dos Santos et al., 2012) | NA | 2012-11-12 |
| Anabaenopsis | 1 | Yes | (Watanabe & Yamamoto, 1967) | NA | 2017-06-24 |
| Anaerobacillus | 2 | Yes | (Zavarzina et al., 2009) | NA | 2016-11-02 |
| Anaerocolumna | 2 | Yes | NA | Previously *Clostridium neopropionicum* | 2016-12-02 |
| Anaeromyxobacter | 4 | Yes | (Masuda et al., 2020) | NA | NA |
| Anaerosporobacter | 1 | No | NA | NA | 2016-12-02 |
| Anaerosporomusa | 1 | Yes | (Suárez-Moo et al., 2020) | NA | 2016-04-04 |
| Anaerotignum | 1 | Yes | NA | Previously *Clostridium neopropionicum* | 2016-02-23 |
| Anaerovirgula | 1 | No | NA | NA | 2017-07-15 |
| Aquaspirillum | 1 | Yes | (Brenner et al., 2007) | NA | 2017-02-16 |
| Arcobacter | 2 | Yes | (Dos Santos et al., 2012) | NA | 2010-05-16 |
| Arcticibacter | 1 | No | NA | NA | 2013-05-28 |
| Aulosira | 1 | Yes | (Issa et al., 2014) | NA | 2017-06-24 |
| Azoarcus | 3 | Yes | (Hurek & Reinhold-Hurek, 2003) | NA | 2006-12-19 |
| Azonexus | 1 | Yes | (Brenner et al., 2007) | NA | 2017-01-17 |
| Azorhizobium | 1 | Yes | (Liu et al., 2017) | NA | 2007-10-15 |
| Azospira | 2 | Yes | (Brenner et al., 2007) | NA | 2011-12-04 |
| Azospirillum | 9 | Yes | (Zhang et al., 2020) | NA | 2010-02-15 |
| Azotobacter | 4 | Yes | (Dos Santos et al., 2012) | NA | 2009-04-13 |
| Bacillus | 2 | No | NA | NA | 2016-10-21 |
| Bacteroides | 2 | Yes | (Inoue et al., 2015) | NA | 2014-03-30 |
| Beggiatoa | 2 | Yes | (Dubinina et al., 2017) | NA | 2012-05-03 |
| Beijerinckia | 1 | Yes | (Dos Santos et al., 2012) | NA | 2008-04-10 |
| Blastochloris | 1 | Yes | NA | Previously *Rhodopseudomonas* | 2016-02-23 |
| Bradyrhizobium | 41 | Yes | (Dos Santos et al., 2012) | NA | 2004-05-10 |
| Brenneria | 2 | No | NA | NA | 2011-10-20 |
| Burkholderia | 8 | Yes | (Dos Santos et al., 2012) | NA | 2007-03-26 |
| Caenispirillum | 2 | No | NA | NA | 2012-11-28 |
| Calditerrivibrio | 1 | No | NA | NA | 2010-11-29 |
| Calothrix | 10 | Yes | (Issa et al., 2014) | NA | 2012-12-03 |
| Candidatus Accumulibacter | 1 | No | NA | NA | NA |
| Candidatus Atelocyanobacterium | 1 | Yes | (Muñoz-Marín et al., 2019) | NA | 2010-01-27 |
| Candidatus Azobacteroides | 1 | Yes | (Hongoh et al., 2008) | NA | 2008-11-11 |
| Candidatus Dactylopiibacterium | 1 | Yes | (Vera-Ponce de León et al., 2017) | NifH transcription evidence | 2017-08-31 |
| Candidatus Magnetoovum | 1 | No | NA | NA | 2015-03-22 |
| Candidatus Thiodiazotropha | 1 | Yes | (König et al., 2016) | NifH transcription evidence | 2016-08-22 |
| Chlorobaculum | 3 | Yes | (Dos Santos et al., 2012) | NA | 2002-07-01 |
| Chlorobium | 5 | Yes | (Dos Santos et al., 2012) | NA | 2005-10-25 |
| Chloroherpeton | 1 | No | NA | NA | 2008-06-26 |
| Chroococcidiopsis | 2 | Yes | (Banerjee & Verma, 2009) | NA | 2012-12-04 |
| Chroogloeocystis | 1 | No | NA | NA | 2016-12-13 |
| Clostridium | 31 | Yes | (Dos Santos et al., 2012) | NA | 2007-06-26 |
| Cohaesibacter | 2 | No | NA | NA | 2016-11-01 |
| Coleofasciculus | 1 | Yes | NA | Previously known as *Microcoelus* | 2008-08-14 |
| Confluentimicrobium | 1 | Metagenome | NA | NA | 2017-10-24 |
| Consotaella | 1 | Yes | (Díaz-Cárdenas et al., 2017) | NA | 2017-03-03 |
| Coraliomargarita | 1 | No | NA | NA | 2010-04-04 |
| Crenothrix | 1 | Metagenome | NA | NA | 2017-05-11 |
| Crocosphaera | 8 | Yes | (Boyd & Peters, 2013) | NA | 2005-06-15 |
| Cupriavidus | 1 | Yes | (Dos Santos et al., 2012) | NA | 2008-03-26 |
| Cyanothece | 1 | Yes | (Dos Santos et al., 2012) | NA | 2009-01-08 |
| Cylindrospermopsis | 3 | Yes | (Willis et al., 2016) | NA | 2009-12-14 |
| Cylindrospermum | 1 | Yes | (Issa et al., 2014) | NA | 2017-06-24 |
| Dechloromonas | 2 | No | NA | NA | 2005-08-04 |
| Defluviimonas | 1 | No | NA | NA | 2016-04-14 |
| Dehalobacter | 2 | No | NA | NA | 2012-10-15 |
| Dehalococcoides | 1 | Yes | (Dos Santos et al., 2012) | NA | 2005-01-06 |
| Dendrosporobacter | 1 | Yes | NA | Was originally *Clostridium* | 2016-10-20 |
| Denitrovibrio | 2 | No | NA | NA | 2010-03-10 |
| Desertifilum | 1 | No | NA | NA | 2016-09-25 |
| Desulfacinum | 1 | No | NA | NA | 2016-12-02 |
| Desulfallas | 3 | Yes | NA | Previously *Desulfotomaculum* | 2013-05-05 |
| Desulfamplus | 1 | No | NA | NA | 2017-03-28 |
| Desulfarculus | 1 | No | NA | NA | 2010-07-28 |
| Desulfatibacillum | 2 | Yes | (Cravo-Laureau et al., 2004) | NA | 2008-12-23 |
| Desulfatitalea | 1 | Metagenome | NA | NA | 2015-03-23 |
| Desulfitobacterium | 1 | Yes | (Dos Santos et al., 2012) | NA | 2009-01-04 |
| Desulfobacca | 1 | No | NA | NA | 2011-04-06 |
| Desulfobacter | 1 | Yes | (Thajudeen et al., 2017) | NA | 2012-05-30 |
| Desulfobacterium | 1 | No | NA | NA | 2009-02-17 |
| Desulfobacula | 1 | Metagenome | NA | NA | 2016-10-19 |
| Desulfobulbus | 1 | Metagenome | NA | NA | 2014-10-27 |
| Desulfocapsa | 1 | Yes | (Finster et al., n.d.) | NA | 2013-02-19 |
| Desulfocarbo | 1 | No | NA | NA | 2015-07-20 |
| Desulfocucumis | 1 | No | NA | NA | 2018-02-06 |
| Desulfocurvibacter | 2 | No | NA | NA | 2012-01-03 |
| Desulfofarcimen | 1 | Yes | NA | Previously *Desulfotomaculum* | 2009-09-09 |
| Desulfofustis | 1 | Yes | (Finster et al., n.d.) | NA | 2016-12-02 |
| Desulfohalovibrio | 1 | Yes | NA | Previously *Desulfovibrio* | 2013-07-10 |
| Desulfoluna | 1 | No | NA | NA | 2016-10-17 |
| Desulfomicrobium | 3 | No | NA | NA | 2009-08-25 |
| Desulfomonile | 1 | No | NA | NA | 2012-06-11 |
| Desulfonatronospira | 1 | No | NA | NA | 2010-06-16 |
| Desulfonatronum | 1 | No | NA | NA | 2016-10-20 |
| Desulfopila | 1 | No | NA | NA | 2016-12-09 |
| Desulforhopalus | 1 | No | NA | NA | 2016-10-20 |
| Desulfosporosinus | 9 | Yes | (Vos et al., 2011) | NA | 2011-08-25 |
| Desulfotalea | 1 | Metagenome | NA | NA | 2017-10-25 |
| Desulfotomaculum | 8 | Yes | (Dos Santos et al., 2012) | NA | 2007-03-23 |
| Desulfovibrio | 13 | Yes | (Dos Santos et al., 2012) | NA | 2004-04-20 |
| Desulfuribacillus | 2 | No | NA | NA | 2016-09-15 |
| Desulfurivibrio | 1 | No | NA | NA | 2010-05-26 |
| Desulfurobacterium | 1 | No | NA | NA | 2017-07-15 |
| Desulfuromonas | 1 | No | NA | NA | 2015-09-01 |
| Dethiobacter | 1 | No | NA | NA | 2009-03-16 |
| Dickeya | 4 | Yes | NA | Previously classified as *Erwinia* | 2009-06-25 |
| Draconibacterium | 1 | No | NA | NA | 2015-03-09 |
| Dysgonomonas | 1 | Yes | (Inoue et al., 2015) | NA | 2011-05-17 |
| Ectothiorhodospira | 4 | Yes | (Rosenberg et al., 2014) | NA | 2016-04-26 |
| Ensifer | 5 | Yes | NA | NA | 2016-05-24 |
| Enterobacter | 2 | No | NA | NA | 2013-06-10 |
| Euhalothece | 1 | Yes | (Mogany et al., 2018) | NA | 2018-01-24 |
| Ferrovum | 1 | Yes | (Ullrich et al., 2016) | NA | 2016-03-07 |
| Fischerella | 11 | Yes | (Issa et al., 2014) | NA | 2011-10-20 |
| Fontibacillus | 1 | No | NA | NA | 2016-10-20 |
| Frankia | 17 | Yes | (Dos Santos et al., 2012) | NA | 2006-08-02 |
| Geoalkalibacter | 2 | Yes | (Zavarzina et al., 2006) | NA | 2015-01-15 |
| Geobacter | 2 | Yes | (Dos Santos et al., 2012) | NA | 2009-01-26 |
| Geofilum | 1 | Yes | (Inoue et al., 2015) | NA | 2015-03-25 |
| Geosporobacter | 1 | No | NA | NA | 2016-10-02 |
| Gloeothece | 2 | Yes | (Issa et al., 2014) | NA | 2008-12-16 |
| Gluconacetobacter | 1 | Yes | (Bertalan et al., 2009) | NA | 2008-10-15 |
| Gracilibacter | 1 | Metagenome | NA | NA | 2016-01-14 |
| Gynuella | 1 | No | NA | NA | 2015-03-03 |
| Halanaerobium | 2 | No | NA | NA | 2016-02-28 |
| Halodesulfovibrio | 1 | Yes | NA | Previously *Desulfovibrio* | NA |
| Halomonas | 1 | No | NA | NA | 2018-01-12 |
| Halorhodospira | 2 | Yes | (Dos Santos et al., 2012) | NA | 2007-01-09 |
| Halothece | 1 | Yes | (Zehr et al., 1997) | NA | 2012-12-06 |
| Hartmannibacter | 1 | Yes | (Suarez et al., 2014) | NA | 2017-10-24 |
| Herbaspirillum | 2 | Yes | (Dos Santos et al., 2012) | NA | 2010-07-01 |
| Hungateiclostridium | 1 | Yes | NA | Previously *Clostridium neopropionicum* | 2011-12-11 |
| Hydrococcus | 1 | No | NA | NA | 2016-12-13 |
| Hydrocoleum | 1 | Yes | (Palińska et al., 2015) | NA | 2017-08-17 |
| Hydrogenophaga | 1 | Yes | NA | Formerly known as *Pseudomonas* | 2017-02-15 |
| Hyphomicrobium | 1 | No | NA | NA | 2011-06-23 |
| Ilyobacter | 1 | No | NA | NA | 2010-10-21 |
| Imhoffiella | 1 | No | NA | NA | 2014-03-03 |
| Insolitispirillum | 1 | Yes | (DeLong et al., 2014) | NA | 2017-01-14 |
| Klebsiella | 10 | Yes | (Dos Santos et al., 2012) | NA | 2011-12-28 |
| Kosakonia | 6 | Yes | (Madhaiyan et al., 2013) | NA | 2016-08-03 |
| Kyrpidia | 1 | No | NA | NA | 2017-12-03 |
| Labilibaculum | 2 | No | NA | NA | 2017-06-05 |
| Lachnoclostridium | 2 | Yes | NA | previously *Clostridium neopropionicum* | 2016-10-30 |
| Leptolyngbya | 6 | Yes | (Shimura et al., 2015) | NA | 2012-12-02 |
| Leptospirillum | 4 | Yes | (Parro & Moreno-Paz, 2004) | NA | 2012-03-29 |
| Leptothrix | 1 | No | NA | NA | 2008-03-24 |
| Limnoraphis | 1 | Yes | (Komárková et al., 2016) | NA | 2015-07-16 |
| Lutibacter | 1 | No | NA | NA | 2017-07-15 |
| Lyngbya | 2 | Yes | (Omoregie et al., 2004) | NA | 2006-12-14 |
| Magnetococcus | 1 | Yes | (Bazylinski et al., 2013) | NA | 2006-11-07 |
| Magnetospirillum | 9 | Yes | (Dos Santos et al., 2012) | NA | 2005-11-30 |
| Magnetovibrio | 1 | Yes | (DeLong et al., 2014) | NA | 2016-09-25 |
| Mangrovibacter | 2 | Yes | (Behera et al., 2017) | NA | 2014-06-11 |
| Marichromatium | 1 | No | NA | NA | 2014-01-02 |
| Marinobacter | 1 | Metagenome | NA | NA | 2013-10-20 |
| Marinobacterium | 2 | Yes | (Alfaro-Espinoza & Ullrich, 2014) |  | 2014-06-11 |
| Marinospirillum | 1 | No | NA | NA | 2016-11-17 |
| Martelella | 1 | Yes | (정영륜 et al., 2013) | NA | 2015-03-23 |
| Mastigocoleus | 1 | Yes | (Guida & Garcia-Pichel, 2016) | NA | 2015-12-02 |
| Mesorhizobium | 66 | Yes | (Dos Santos et al., 2012) | NA | 2004-05-10 |
| Methanobacterium | 4 | Yes | (Dos Santos et al., 2012) | NA | 2013-11-13 |
| Methanocella | 2 | No | NA | NA | 2006-10-11 |
| Methanococcus | 4 | Yes | (Dos Santos et al., 2012) | NA | 2007-03-14 |
| Methanolacinia | 1 | No | NA | NA | 2010-09-16 |
| Methanolobus | 2 | No | NA | NA | 2016-02-28 |
| Methanoregula | 1 | No | NA | NA | 2007-07-25 |
| Methanosarcina | 24 | Yes | (Dos Santos et al., 2012) | NA | 2002-04-02 |
| Methanosphaerula | 1 | No | NA | NA | 2009-01-04 |
| Methanothermobacter | 4 | Yes | (Dos Santos et al., 2012) | NA | 1999-12-21 |
| Methanothrix | 1 | Yes | (Bae et al., 2018) | NifH transcription evidence | 2011-04-14 |
| Methylacidiphilum | 4 | Yes | (Khadem et al., 2010) | NA | 2008-05-29 |
| Methylobacter | 2 | No | NA | NA | 2011-08-24 |
| Methylobacterium | 2 | Yes | (Dos Santos et al., 2012) | NA | 2008-02-19 |
| Methylocaldum | 1 | No | NA | NA | 2017-09-07 |
| Methylocapsa | 1 | Yes | (Dedysh et al., 2002) | NA | 2016-11-01 |
| Methyloceanibacter | 1 | No | NA | NA | 2016-09-11 |
| Methylocella | 2 | Yes | (Dos Santos et al., 2012) | NA | 2008-12-14 |
| Methylococcus | 1 | Yes | (Dos Santos et al., 2012) | NA | 2005-05-17 |
| Methylocystis | 3 | Yes | (Belova et al., 2013) | NA | 2012-08-19 |
| Methyloglobulus | 1 | Yes | (Deutzmann et al., 2014) | NA | 2013-11-12 |
| Methylomagnum | 1 | No | NA | NA | 2017-04-18 |
| Methylomonas | 7 | Yes | (Dos Santos et al., 2012) | NA | 2011-05-23 |
| Methyloprofundus | 1 | Yes | (Tavormina et al., 2015) |  | 2017-03-27 |
| Methylosinus | 3 | Yes | (Auman et al., 2001) | NA | 2016-05-15 |
| Methyloversatilis | 1 | No | NA | NA | 2016-08-24 |
| Methylovulum | 1 | Yes | (Oshkin et al., 2016) | NA | 2017-06-26 |
| Microchaete | 1 | Yes | (Busch & Montgomery, 2017) | NA | 2017-06-24 |
| Microcoleus | 1 | Yes | (Omoregie et al., 2004) | NA | 2012-12-05 |
| Microvirga | 2 | Yes | (Ardley et al., 2012) | NA | 2012-05-28 |
| Neorhizobium | 4 | Yes | NA | NA | 2014-06-24 |
| Neosynechococcus | 1 | No | NA | NA | 2014-09-28 |
| Nitrospirillum | 2 | Yes | NA | Previously *Azospirillum* | 2011-09-14 |
| Nodularia | 2 | Yes | (Issa et al., 2014) | NA | 2014-04-02 |
| Nostoc | 20 | Yes | (Dos Santos et al., 2012) | NA | 2004-05-10 |
| Novosphingobium | 5 | Yes | (DeLong et al., 2014) | NA | 2011-03-17 |
| Oleibacter | 1 | Metagenome | NA | NA | 2017-10-23 |
| Orenia | 1 | No | NA | NA | 2016-07-25 |
| Paenibacillus | 33 | Yes | NA | NA | 2011-11-15 |
| Paludibacter | 2 | Yes | (Inoue et al., 2015) | NA | 2010-11-22 |
| Pantoea | 3 | Yes | (Dos Santos et al., 2012) | NA | 2011-01-03 |
| Paraburkholderia | 13 | Yes | (Sawana et al., 2014) | NA | 2006-04-09 |
| Pararhodospirillum | 1 | Yes | (DeLong et al., 2014) | NA | 2012-02-22 |
| Pectobacterium | 3 | Yes | NA | Previously classified as *Erwinia* | 2004-06-30 |
| Pelobacter | 1 | Yes | (Dos Santos et al., 2012) | NA | 2006-03-29 |
| Pelodictyon | 2 | Yes | NA | Previously known as *Chlorobium* | 2005-10-25 |
| Pelosinus | 4 | No | NA | NA | 2012-06-19 |
| Phaeospirillum | 2 | Yes | (DeLong et al., 2014) | NA | 2012-03-15 |
| Phormidesmis | 1 | Metagenome | NA | NA | 2015-10-13 |
| Phormidium | 2 | Yes | (Zehr et al., 1997) | NA | 2016-05-04 |
| Phytobacter | 1 | Yes | (Pillonetto et al., 2018) | NA | 2015-06-08 |
| Planktothrix | 1 | Yes | (Pancrace et al., 2017) | NA | 2016-12-01 |
| Pleomorphomonas | 2 | Yes | (Madhaiyan et al., 2013) | NA | 2017-11-13 |
| Pleurocapsa | 1 | Yes | (Bergman et al., 1997) | NA | 2012-12-04 |
| Polaromonas | 1 | Yes | (Inoue et al., 2015) | NA | 2007-01-03 |
| Propionibacterium | 1 | Yes | (Sellstedt & Richau, 2013) | NA | 2016-10-30 |
| Propionispora | 3 | Yes | (Addo & Dos Santos, 2020) | NA | 2015-09-13 |
| Propionivibrio | 2 | No | NA | NA | 2016-06-15 |
| Prosthecochloris | 3 | Yes | (Dos Santos et al., 2012) | NA | 2008-07-17 |
| Prosthecomicrobium | 1 | No | NA | NA | 2015-09-30 |
| Pseudacidovorax | 1 | Yes | (Kämpfer et al., 2008) | NA | 2013-07-30 |
| Pseudanabaena | 1 | Yes | (Zehr et al., 1997) | NA | 2017-08-14 |
| Pseudoclostridium | 1 | Yes | NA | Previously *Clostridium neopropionicum* | 2018-01-23 |
| Pseudodesulfovibrio | 5 | Yes | NA | Previously known as *Desulfovibrio* | 2011-01-03 |
| Pseudomonas | 10 | Yes | (Dos Santos et al., 2012) | NA | 2007-04-19 |
| Rahnella | 3 | Yes | (Berge et al., 1991) | NA | 2012-01-09 |
| Raoultella | 1 | Yes | NA | Previously classified as *Klebsiella* | 2018-12-06 |
| Rhizobium | 46 | Yes | (Dos Santos et al., 2012) | NA | 2006-03-05 |
| Rhodobacter | 13 | Yes | (Dos Santos et al., 2012) | NA | 2007-02-22 |
| Rhodoblastus | 1 | Yes | (Dos Santos et al., 2012) | Previously *Rhodopseudomonas* | 2017-06-23 |
| Rhodoferax | 2 | Yes | (Baker et al., 2017) | NA | 2017-01-05 |
| Rhodomicrobium | 2 | Yes | (Dos Santos et al., 2012) | NA | 2010-11-04 |
| Rhodopseudomonas | 10 | Yes | (Dos Santos et al., 2012) | NA | 2003-12-10 |
| Rhodospira | 1 | No | NA | NA | 2016-10-20 |
| Rhodospirillum | 2 | Yes | (Dos Santos et al., 2012) | NA | 2005-12-11 |
| Rhodovulum | 4 | Yes | NA | Previously *Rhodobacter* | 2015-03-17 |
| Richelia | 1 | Yes | (Hilton et al., 2013) | NA | 2013-03-26 |
| Rippkaea | 1 | Yes | (Mareš et al., 2019) | NA | 2008-12-16 |
| Roseofilum | 1 | No | NA | NA | 2016-12-06 |
| Roseospirillum | 1 | No | NA | NA | 2016-10-20 |
| Rubrivivax | 2 | Yes | (Brenner et al., 2007) | NA | 2011-02-22 |
| Ruminiclostridium | 3 | Yes | NA | Previously *Clostridium neopropionicum* | 2011-03-13 |
| Saccharicrinis | 1 | Yes | (Inoue et al., 2015) | NA | 2014-02-26 |
| Sagittula | 1 | Yes | (Martínez‐Pérez et al., 2018) |  | 2017-12-06 |
| Scytonema | 3 | Yes | (Zehr et al., 1997) | NA | 2017-06-24 |
| Sedimenticola | 1 | No | NA | NA | 2015-05-14 |
| Sediminispirochaeta | 1 | Yes | (Shivani et al., 2016) | NA | 2010-07-28 |
| Serratia | 1 | Yes | (Gyaneshwar et al., 2001) | NA | 2018-01-03 |
| Shewanella | 1 | No | NA | NA | 2014-09-09 |
| Sideroxydans | 2 | No | NA | NA | 2010-03-25 |
| Sinorhizobium | 15 | Yes | (Dos Santos et al., 2012) | NA | 2003-05-05 |
| Skermanella | 2 | No | NA | NA | 2014-02-23 |
| Solimonas | 1 | No | NA | NA | 2016-10-30 |
| Sphaerotilus | 1 | No | NA | NA | 2014-05-11 |
| Spirochaeta | 1 | Yes | (Lilburn et al., 2001) | NA | 2010-09-09 |
| Sporomusa | 4 | Yes | (Kuhner et al., 1997) | NA | 2015-04-19 |
| Sulfuricella | 1 | No | NA | NA | 2015-04-06 |
| Sulfuricurvum | 5 | No | NA | NA | 2010-12-01 |
| Sulfurimonas | 2 | Metagenome | NA | NA | 2016-10-20 |
| Sulfurospirillum | 4 | No | NA | NA | 2012-06-06 |
| Synechococcus | 4 | Yes | (Dos Santos et al., 2012) | NA | 2006-02-05 |
| Syntrophobacter | 1 | No | NA | NA | 2006-10-25 |
| Syntrophobotulus | 1 | No | NA | NA | 2011-02-28 |
| Syntrophomonas | 1 | No | NA | NA | 2015-04-23 |
| Syntrophus | 1 | No | NA | NA | 2016-10-30 |
| Telmatospirillum | 1 | Yes | (DeLong et al., 2014) | NA | 2017-12-19 |
| Teredinibacter | 1 | Yes | (Dos Santos et al., 2012) | NA | 2009-06-14 |
| Terrimicrobium | 1 | No | NA | NA | 2016-03-29 |
| Thermincola | 2 | No | NA | NA | 2010-05-12 |
| Thermoanaerobacterium | 1 | Yes | (Klipp et al., 2005) | Previously *C. thermosaccharolyticum* | 2012-06-03 |
| Thermoclostridium | 1 | Yes | NA | Previously *Clostridium neopropionicum* | 2019-09-10 |
| Thermodesulfovibrio | 2 | No | NA | NA | 2008-09-25 |
| Thiocapsa | 3 | No | NA | NA | 2011-08-17 |
| Thiocystis | 1 | Yes | (DeLong et al., 2014) |  | 2012-06-07 |
| Thiodictyon | 1 | Yes | (Luedin et al., 2019) | NA | 2017-12-06 |
| Thioflavicoccus | 1 | Yes | (DeLong et al., 2014) | NA | 2012-12-19 |
| Thioflexothrix | 1 | No | NA | NA | 2017-05-18 |
| Thioploca | 1 | Metagenome | NA | NA | 2014-09-18 |
| Thiorhodococcus | 1 | Yes | (Addo & Dos Santos, 2020) | NA | 2011-08-18 |
| Thiorhodospira | 1 | Yes | (Severin et al., 2010) | NA | 2011-10-05 |
| Thiorhodovibrio | 1 | Yes | (Rosenberg et al., 2014) | NA | 2012-03-22 |
| Thiothrix | 1 | Yes | (Chernousova et al., 2009) | NA | 2016-10-21 |
| Tolumonas | 1 | No | NA | NA | 2009-05-12 |
| Tolypothrix | 3 | Yes | (Issa et al., 2014) | NA | 2012-09-27 |
| Treponema | 2 | Yes | (Lilburn et al., 2001) | NA | 2011-05-22 |
| Trichodesmium | 1 | Yes | (Dos Santos et al., 2012) | NA | 2006-07-05 |
| Trichormus | 3 | Yes | (Gladkikh et al., 2008) | NA | 2005-09-14 |
| Trinickia | 2 | Yes | (Estrada-de Los Santos et al., 2018) | NA | 2017-04-18 |
| Tropicimonas | 1 | No | NA | NA | 2016-11-01 |
| Uliginosibacterium | 1 | Yes | NA | Previously known as *Azoarcus* | 2018-01-02 |
| Unclassified | 62 | NA | NA | NA | NA |
| Variovorax | 1 | No | NA | NA | 2016-08-15 |
| Vibrio | 10 | Yes | (Dos Santos et al., 2012) | NA | 2016-07-05 |
| Vitreoscilla | 1 | Yes | (Brenner et al., 2007) | NA | 2017-07-19 |
| Wolinella | 1 | Yes | NA | Previously known as *Vibrio* | 2003-09-07 |
| Xanthobacter | 1 | Yes | (Dos Santos et al., 2012) | NA | 2007-07-29 |
| Xenococcus | 1 | Yes | (Zehr et al., 1997) | NA | 2013-01-16 |
| Yangia | 2 | No | NA | NA | 2016-07-17 |
| Youngiibacter | 1 | No | NA | NA | 2013-12-08 |
| Zymomonas | 4 | Yes | (Kremer et al., 2015) | NA | 2010-01-11 |

References:

Addo, M. A., & Dos Santos, P. C. (2020). Distribution of Nitrogen-Fixation Genes in Prokaryotes Containing Alternative Nitrogenases. *ChemBioChem*, *21*(12), 1749–1759. https://doi.org/10.1002/cbic.202000022

Alfaro-Espinoza, G., & Ullrich, M. S. (2014). Marinobacterium mangrovicola sp. Nov., a marine nitrogen-fixing bacterium isolated from mangrove roots of Rhizophora mangle. *International Journal of Systematic and Evolutionary Microbiology*, *64*(Pt_12), 3988–3993. https://doi.org/10.1099/ijs.0.067462-0

Ardley, J. K., Parker, M. A., De Meyer, S. E., Trengove, R. D., O’Hara, G. W., Reeve, W. G., Yates, R. J., Dilworth, M. J., Willems, A., & Howieson, J. G. (2012). Microvirga lupini sp. Nov., Microvirga lotononidis sp. Nov., and Microvirga zambiensis sp. Nov. Are Alphaproteobacterial root nodule bacteria that specifically nodulate and fix nitrogen with geographically and taxonomically separate legume hosts. *International Journal of Systematic and Evolutionary Microbiology*, *62*(11), 2579–2588.

Auman, A. J., Speake, C. C., & Lidstrom, M. E. (2001). NifH Sequences and Nitrogen Fixation in Type I and Type II Methanotrophs. *Applied and Environmental Microbiology*, *67*(9), 4009–4016. https://doi.org/10.1128/AEM.67.9.4009-4016.2001

Bae, H.-S., Morrison, E., Chanton, J. P., & Ogram, A. (2018). Methanogens Are Major Contributors to Nitrogen Fixation in Soils of the Florida Everglades. *Applied and Environmental Microbiology*, *84*(7). https://doi.org/10.1128/AEM.02222-17

Baker, J. M., Riester, C. J., Skinner, B. M., Newell, A. W., Swingley, W. D., Madigan, M. T., Jung, D. O., Asao, M., Chen, M., Loughlin, P. C., Pan, H., Lin, Y., Li, Y., Shaw, J., Prado, M., Sherman, C., Tang, J. K.-H., Blankenship, R. E., Zhao, T., … Sattley, W. M. (2017). Genome Sequence of Rhodoferax antarcticus ANT.BRT; A Psychrophilic Purple Nonsulfur Bacterium from an Antarctic Microbial Mat. *Microorganisms*, *5*(1). https://doi.org/10.3390/microorganisms5010008

Banerjee, M., & Verma, V. (2009). Nitrogen fixation in endolithic cyanobacterial communities of the McMurdo Dry Valley, Antarctica. *ScienceAsia*, *35*(3), 215. https://doi.org/10.2306/scienceasia1513-1874.2009.35.215

Bazylinski, D. A., Williams, T. J., Lefevre, C. T., Berg, R. J., Zhang, C. L., Bowser, S. S., Dean, A. J., & Beveridge, T. J. (2013). Magnetococcus marinus gen. Nov., sp. Nov., a marine, magnetotactic bacterium that represents a novel lineage (Magnetococcaceae fam. Nov., Magnetococcales ord. Nov.) at the base of the Alphaproteobacteria. *INTERNATIONAL JOURNAL OF SYSTEMATIC AND EVOLUTIONARY MICROBIOLOGY*, *63*(Pt 3), 801–808. https://doi.org/10.1099/ijs.0.038927-0

Behera, P., Venkata Ramana, V., Maharana, B., Joseph, N., Vaishampayan, P., Singh, N. K., Shouche, Y., Bhadury, P., Mishra, S. R., Raina, V., Suar, M., Pattnaik, A. K., & Rastogi, G. (2017). Mangrovibacter phragmitis sp. Nov., an endophyte isolated from the roots of Phragmites karka. *International Journal of Systematic and Evolutionary Microbiology*, *67*(5), 1228–1234. https://doi.org/10.1099/ijsem.0.001789

Belova, S. E., Kulichevskaya, I. S., Bodelier, P. L. E., & Dedysh, S. N. (2013). Methylocystis bryophila sp. Nov., a facultatively methanotrophic bacterium from acidic Sphagnum peat, and emended description of the genus Methylocystis (ex Whittenbury et al. 1970) Bowman et. *International Journal of Systematic and Evolutionary Microbiology*, *63*(Pt_3), 1096–1104. https://doi.org/10.1099/ijs.0.043505-0

Berge, O., Heulin, T., Achouak, W., Richard, C., Bally, R., & Balandreau, J. (1991). Rahnella aquatilis, a nitrogen-fixing enteric bacterium associated with the rhizosphere of wheat and maize. *Canadian Journal of Microbiology*, *37*(3), 195–203. https://doi.org/10.1139/m91-030

Bergman, B., Gallon, J. R., Rai, A. N., & Stal, L. J. (1997). N2 Fixation by non-heterocystous cyanobacteria. *FEMS Microbiology Reviews*, *19*(3), 139–185. https://doi.org/10.1111/j.1574-6976.1997.tb00296.x

Bertalan, M., Albano, R., de Pádua, V., Rouws, L., Rojas, C., Hemerly, A., Teixeira, K., Schwab, S., Araujo, J., Oliveira, A., França, L., Magalhães, V., Alquéres, S., Cardoso, A., Almeida, W., Loureiro, M. M., Nogueira, E., Cidade, D., Oliveira, D., … Ferreira, P. C. (2009). Complete genome sequence of the sugarcane nitrogen-fixing endophyte Gluconacetobacter diazotrophicus Pal5. *BMC Genomics*, *10*(1), 450. https://doi.org/10.1186/1471-2164-10-450

Boyd, E., & Peters, J. W. (2013). New insights into the evolutionary history of biological nitrogen fixation. *Frontiers in Microbiology*, *4*. https://doi.org/10.3389/fmicb.2013.00201

Brenner, D. J., Krieg, N. R., & Staley, J. R. (2007). *Bergey’s Manual® of Systematic Bacteriology: Volume 2: The Proteobacteria, Part B: The Gammaproteobacteria*. Springer Science & Business Media.

Busch, A. W. U., & Montgomery, B. L. (2017). Distinct light-, stress-, and nutrient-dependent regulation of multiple tryptophan-rich sensory protein (TSPO) genes in the cyanobacterium Fremyella diplosiphon. *Plant Signaling & Behavior*, *12*(3). https://doi.org/10.1080/15592324.2017.1293221

Chernousova, E., Gridneva, E., Grabovich, M., Dubinina, G., Akimov, V., Rossetti, S., & Kuever, J. (2009). Thiothrix caldifontis sp. Nov. And Thiothrix lacustris sp. Nov., gammaproteobacteria isolated from sulfide springs. *International Journal of Systematic and Evolutionary Microbiology*, *59*(Pt 12), 3128–3135. https://doi.org/10.1099/ijs.0.009456-0

Cravo-Laureau, C., Matheron, R., Cayol, J.-L., Joulian, C., & Hirschler-Réa, A. (2004). Desulfatibacillum aliphaticivorans gen. Nov., sp. Nov., an n-alkane- and n-alkene-degrading, sulfate-reducing bacterium. *International Journal of Systematic and Evolutionary Microbiology*, *54*(Pt 1), 77–83. https://doi.org/10.1099/ijs.0.02717-0

Dedysh, S. N., Khmelenina, V. N., Suzina, N. E., Trotsenko, Y. A., Semrau, J. D., Liesack, W., & Tiedje, J. M. (2002). Methylocapsa acidiphila gen. Nov., sp. Nov., a novel methane-oxidizing and dinitrogen-fixing acidophilic bacterium from Sphagnum bog. *International Journal of Systematic and Evolutionary Microbiology*, *52*(Pt 1), 251–261. https://doi.org/10.1099/00207713-52-1-251

DeLong, E. F., Lory, S., Stackebrandt, E., & Thompson, F. (2014). *The Prokaryotes: Alphaproteobacteria and Betaproteobacteria*. Springer Berlin Heidelberg.

Deutzmann, J. S., Hoppert, M., & Schink, B. (2014). Characterization and phylogeny of a novel methanotroph, Methyloglobulus morosus gen. Nov., spec. Nov. *Systematic and Applied Microbiology*, *37*(3), 165–169. https://doi.org/10.1016/j.syapm.2014.02.001

Díaz-Cárdenas, C., Bernal, L. F., Caro-Quintero, A., López, G., David Alzate, J., Gonzalez, L. N., Restrepo, S., Shapiro, N., Woyke, T., Kyrpides, N. C., & Baena, S. (2017). Draft genome and description of Consotaella salsifontis gen. Nov. Sp. Nov., a halophilic, free-living, nitrogen-fixing alphaproteobacterium isolated from an ancient terrestrial saline spring. *International Journal of Systematic and Evolutionary Microbiology*, *67*(10), 3744–3751. https://doi.org/10.1099/ijsem.0.002185

Dos Santos, P. C., Fang, Z., Mason, S. W., Setubal, J. C., & Dixon, R. (2012). Distribution of nitrogen fixation and nitrogenase-like sequences amongst microbial genomes. *BMC Genomics*, *13*(1), 162. https://doi.org/10.1186/1471-2164-13-162

Dubinina, G., Savvichev, A., Orlova, M., Gavrish, E., Verbarg, S., & Grabovich, M. (2017). Beggiatoa leptomitoformis sp. Nov., the first freshwater member of the genus capable of chemolithoautotrophic growth. *International Journal of Systematic and Evolutionary Microbiology*, *67*(2), 197–204. https://doi.org/10.1099/ijsem.0.001584

Estrada-de Los Santos, P., Palmer, M., Chávez-Ramírez, B., Beukes, C., Steenkamp, E. T., Briscoe, L., Khan, N., Maluk, M., Lafos, M., Humm, E., Arrabit, M., Crook, M., Gross, E., Simon, M. F., Dos Reis Junior, F. B., Whitman, W. B., Shapiro, N., Poole, P. S., Hirsch, A. M., … James, E. K. (2018). Whole Genome Analyses Suggests that Burkholderia sensu lato Contains Two Additional Novel Genera (Mycetohabitans gen. nov., and Trinickia gen. nov.): Implications for the Evolution of Diazotrophy and Nodulation in the Burkholderiaceae. *Genes*, *9*(8). https://doi.org/10.3390/genes9080389

Finster, K. W., Kjeldsen, K. U., Kube, M., Reinhardt, R., Mussmann, M., Amann, R., & Schreiber, L. (n.d.). Complete genome sequence of Desulfocapsa sulfexigens, a marine deltaproteobacterium specialized in disproportionating inorganic sulfur compounds. *Standards in Genomic Sciences*, *8*(1), 58–68.

Gladkikh, A. S., Belykh, O. I., Klimenkov, I. V., & Tikhonova, I. V. (2008). [Nitrogen-fixing cyanobacterium Trichormus variabilis of the lake Baikal phytoplankton]. *Mikrobiologiia*, *77*(6), 814–822.

Guida, B. S., & Garcia-Pichel, F. (2016). Draft Genome Assembly of a Filamentous Euendolithic (True Boring) Cyanobacterium, *Mastigocoleus testarum* Strain BC008. *Genome Announcements*, *4*(1), e01574-15, /ga/4/1/e01574-15.atom. https://doi.org/10.1128/genomeA.01574-15

Gyaneshwar, P., James, E. K., Mathan, N., Reddy, P. M., Reinhold-Hurek, B., & Ladha, J. K. (2001). Endophytic Colonization of Rice by a Diazotrophic Strain of Serratia marcescens. *Journal of Bacteriology*, *183*(8), 2634–2645. https://doi.org/10.1128/JB.183.8.2634-2645.2001

Hilton, J. A., Foster, R. A., Tripp, H. J., Carter, B. J., Zehr, J. P., & Villareal, T. A. (2013). Genomic deletions disrupt nitrogen metabolism pathways of a cyanobacterial diatom symbiont. *Nature Communications*, *4*(1), 1–7. https://doi.org/10.1038/ncomms2748

Hongoh, Y., Sharma, V. K., Prakash, T., Noda, S., Toh, H., Taylor, T. D., Kudo, T., Sakaki, Y., Toyoda, A., Hattori, M., & Ohkuma, M. (2008). Genome of an Endosymbiont Coupling N2 Fixation to Cellulolysis Within Protist Cells in Termite Gut. *Science*, *322*(5904), 1108–1109. https://doi.org/10.1126/science.1165578

Hurek, T., & Reinhold-Hurek, B. (2003). Azoarcus sp. Strain BH72 as a model for nitrogen-fixing grass endophytes. *Journal of Biotechnology*, *106*(2–3), 169–178. https://doi.org/10.1016/j.jbiotec.2003.07.010

Inoue, J., Oshima, K., Suda, W., Sakamoto, M., Iino, T., Noda, S., Hongoh, Y., Hattori, M., & Ohkuma, M. (2015). Distribution and Evolution of Nitrogen Fixation Genes in the Phylum Bacteroidetes. *Microbes and Environments*, *30*(1), 44–50. https://doi.org/10.1264/jsme2.ME14142

Issa, A. A., Abd-Alla, M. H., & Ohyama, T. (2014). Nitrogen Fixing Cyanobacteria: Future Prospect. *Advances in Biology and Ecology of Nitrogen Fixation*. https://doi.org/10.5772/56995

Kämpfer, P., Thummes, K., Chu, H.-I., Tan, C.-C., Arun, A. B., Chen, W.-M., Lai, W.-A., Shen, F.-T., Rekha, P. D., & Young, C.-C. (2008). Pseudacidovorax intermedius gen. Nov., sp. Nov., a novel nitrogen-fixing betaproteobacterium isolated from soil. *International Journal of Systematic and Evolutionary Microbiology*, *58*(Pt 2), 491–495. https://doi.org/10.1099/ijs.0.65175-0

Khadem, A. F., Pol, A., Jetten, M. S. M., & Op den Camp, H. J. M. (2010). Nitrogen fixation by the verrucomicrobial methanotroph “Methylacidiphilum fumariolicum” SolV. *Microbiology (Reading, England)*, *156*(Pt 4), 1052–1059. https://doi.org/10.1099/mic.0.036061-0

Klipp, W., Masepohl, B., Gallon, J. R., & Newton, W. E. (Eds.). (2005). *Genetics and Regulation of Nitrogen Fixation in Free-Living Bacteria* (Vol. 2). Kluwer Academic Publishers. https://doi.org/10.1007/1-4020-2179-8

Komárková, J., Montoya, H., & Komárek, J. (2016). Cyanobacterial water bloom of Limnoraphis robusta in the Lago Mayor of Lake Titicaca. Can it develop? *Hydrobiologia*, *764*(1), 249–258. https://doi.org/10.1007/s10750-015-2298-x

König, S., Gros, O., Heiden, S. E., Hinzke, T., Thürmer, A., Poehlein, A., Meyer, S., Vatin, M., Mbéguié-A-Mbéguié, D., Tocny, J., Ponnudurai, R., Daniel, R., Becher, D., Schweder, T., & Markert, S. (2016). Nitrogen fixation in a chemoautotrophic lucinid symbiosis. *Nature Microbiology*, *2*(1), 1–10. https://doi.org/10.1038/nmicrobiol.2016.193

Kremer, T. A., LaSarre, B., Posto, A. L., & McKinlay, J. B. (2015). N2 gas is an effective fertilizer for bioethanol production by Zymomonas mobilis. *Proceedings of the National Academy of Sciences*, *112*(7), 2222–2226. https://doi.org/10.1073/pnas.1420663112

Kuhner, C. H., Frank, C., GRIEssZHAMMER, A., Schmittroth, M., Acker, G., GossZNER, A., & Drake, H. L. (1997). Sporomusa silvacetica sp. Nov., an Acetogenic Bacterium Isolated from Aggregated Forest Soil. *International Journal of Systematic Bacteriology*, *47*(2), 352–358. https://doi.org/10.1099/00207713-47-2-352

Lilburn, T. G., Kim, K. S., Ostrom, N. E., Byzek, K. R., Leadbetter, J. R., & Breznak, J. A. (2001). Nitrogen Fixation by Symbiotic and Free-Living Spirochetes. *Science*, *292*(5526), 2495–2498. JSTOR.

Liu, W., Yang, J., Sun, Y., Liu, X., Li, Y., Zhang, Z., & Xie, Z. (2017). Azorhizobium caulinodans Transmembrane Chemoreceptor TlpA1 Involved in Host Colonization and Nodulation on Roots and Stems. *Frontiers in Microbiology*, *8*. https://doi.org/10.3389/fmicb.2017.01327

Luedin, S. M., Storelli, N., Danza, F., Roman, S., Wittwer, M., Pothier, J. F., & Tonolla, M. (2019). Mixotrophic Growth Under Micro-Oxic Conditions in the Purple Sulfur Bacterium “Thiodictyon syntrophicum.” *Frontiers in Microbiology*, *10*. https://doi.org/10.3389/fmicb.2019.00384

Madhaiyan, M., Jin, T. Y., Roy, J. J., Kim, S.-J., Weon, H.-Y., Kwon, S.-W., & Ji, L. (2013). Pleomorphomonas diazotrophica sp. Nov., an endophytic N-fixing bacterium isolated from root tissue of Jatropha curcas L. *International Journal of Systematic and Evolutionary Microbiology*, *63*(Pt 7), 2477–2483. https://doi.org/10.1099/ijs.0.044461-0

Madigan, M. T. (1995). Microbiology of Nitrogen Fixation by Anoxygenic Photosynthetic Bacteria. In R. E. Blankenship, M. T. Madigan, & C. E. Bauer (Eds.), *Anoxygenic Photosynthetic Bacteria* (pp. 915–928). Springer Netherlands. https://doi.org/10.1007/0-306-47954-0_42

Mareš, J., Johansen, J. R., Hauer, T., Zima, J., Ventura, S., Cuzman, O., Tiribilli, B., & Kaštovský, J. (2019). Taxonomic resolution of the genus Cyanothece (Chroococcales, Cyanobacteria), with a treatment on Gloeothece and three new genera, Crocosphaera, Rippkaea, and Zehria. *Journal of Phycology*, *55*(3), 578–610. https://doi.org/10.1111/jpy.12853

Martínez‐Pérez, C., Mohr, W., Schwedt, A., Dürschlag, J., Callbeck, C. M., Schunck, H., Dekaezemacker, J., Buckner, C. R. T., Lavik, G., Fuchs, B. M., & Kuypers, M. M. M. (2018). Metabolic versatility of a novel N2-fixing Alphaproteobacterium isolated from a marine oxygen minimum zone. *Environmental Microbiology*, *20*(2), 755–768. https://doi.org/10.1111/1462-2920.14008

Masuda, Y., Yamanaka, H., Xu, Z.-X., Shiratori, Y., Aono, T., Amachi, S., Senoo, K., & Itoh, H. (2020). Diazotrophic Anaeromyxobacter Isolates from Soils. *Applied and Environmental Microbiology*, *86*(16). https://doi.org/10.1128/AEM.00956-20

Mogany, T., Swalaha, F. M., Allam, M., Mtshali, P. S., Ismail, A., Kumari, S., & Bux, F. (2018). Phenotypic and genotypic characterisation of an unique indigenous hypersaline unicellular cyanobacterium, Euhalothece sp.nov. *Microbiological Research*, *211*, 47–56. https://doi.org/10.1016/j.micres.2018.04.001

Muñoz-Marín, M. del C., Shilova, I. N., Shi, T., Farnelid, H., Cabello, A. M., & Zehr, J. P. (2019). The Transcriptional Cycle Is Suited to Daytime N2 Fixation in the Unicellular Cyanobacterium “Candidatus Atelocyanobacterium thalassa” (UCYN-A). *MBio*, *10*(1). https://doi.org/10.1128/mBio.02495-18

Omoregie, E. O., Crumbliss, L. L., Bebout, B. M., & Zehr, J. P. (2004). Determination of Nitrogen-Fixing Phylotypes in Lyngbya sp. And Microcoleus chthonoplastes Cyanobacterial Mats from Guerrero Negro, Baja California, Mexico. *Applied and Environmental Microbiology*, *70*(4), 2119–2128. https://doi.org/10.1128/AEM.70.4.2119-2128.2004

Oshkin, I. Y., Belova, S. E., Danilova, O. V., Miroshnikov, K. K., Rijpstra, W. I. C., Sinninghe Damsté, J. S., Liesack, W., & Dedysh, S. N. (2016). Methylovulum psychrotolerans sp. Nov., a cold-adapted methanotroph from low-temperature terrestrial environments, and emended description of the genus Methylovulum. *International Journal of Systematic and Evolutionary Microbiology*, *66*(6), 2417–2423. https://doi.org/10.1099/ijsem.0.001046

Palińska, K. A., Abed, R. M. M., Charpy, L., Langlade, M.-J., Beltrán-Magos, Y., & Golubic, S. (2015). Morphological, genetic and physiological characterization of *Hydrocoleum* , the most common benthic cyanobacterium in tropical oceans. *European Journal of Phycology*, *50*(2), 139–154. https://doi.org/10.1080/09670262.2015.1010239

Pancrace, C., Barny, M.-A., Ueoka, R., Calteau, A., Scalvenzi, T., Pédron, J., Barbe, V., Piel, J., Humbert, J.-F., & Gugger, M. (2017). Insights into the Planktothrix genus: Genomic and metabolic comparison of benthic and planktic strains. *Scientific Reports*, *7*. https://doi.org/10.1038/srep41181

Parro, V., & Moreno-Paz, M. (2004). Nitrogen fixation in acidophile iron-oxidizing bacteria: The nif regulon of Leptospirillum ferrooxidans. *Research in Microbiology*, *155*(9), 703–709. https://doi.org/10.1016/j.resmic.2004.05.010

Pillonetto, M., Arend, L. N., Faoro, H., D’Espindula, H. R. S., Blom, J., Smits, T. H. M., Mira, M. T., & Rezzonico, F. (2018). Emended description of the genus Phytobacter, its type species Phytobacter diazotrophicus (Zhang 2008) and description of Phytobacter ursingii sp. Nov. *International Journal of Systematic and Evolutionary Microbiology*, *68*(1), 176–184. https://doi.org/10.1099/ijsem.0.002477

Rosenberg, E., DeLong, E. F., Lory, S., Stackebrandt, E., & Thompson, F. (Eds.). (2014). *The Prokaryotes: Gammaproteobacteria* (4th ed.). Springer-Verlag. https://www.springer.com/gp/book/9783642389214

Sawana, A., Adeolu, M., & Gupta, R. S. (2014). Molecular signatures and phylogenomic analysis of the genus Burkholderia: Proposal for division of this genus into the emended genus Burkholderia containing pathogenic organisms and a new genus Paraburkholderia gen. nov. harboring environmental species. *Frontiers in Genetics*, *5*, 429. https://doi.org/10.3389/fgene.2014.00429

Sellstedt, A., & Richau, K. H. (2013). Aspects of nitrogen-fixing Actinobacteria, in particular free-living and symbiotic Frankia. *FEMS Microbiology Letters*, *342*(2), 179–186. https://doi.org/10.1111/1574-6968.12116

Severin, I., Acinas, S. G., & Stal, L. J. (2010). Diversity of nitrogen-fixing bacteria in cyanobacterial mats: Diversity of diazotrophs in microbial mats. *FEMS Microbiology Ecology*, no-no. https://doi.org/10.1111/j.1574-6941.2010.00925.x

Shimura, Y., Hirose, Y., Misawa, N., Osana, Y., Katoh, H., Yamaguchi, H., & Kawachi, M. (2015). Comparison of the terrestrial cyanobacterium Leptolyngbya sp. NIES-2104 and the freshwater Leptolyngbya boryana PCC 6306 genomes. *DNA Research: An International Journal for Rapid Publication of Reports on Genes and Genomes*, *22*(6), 403–412. https://doi.org/10.1093/dnares/dsv022

Shivani, Y., Subhash, Y., Sasikala, C., & Ramana, C. V. (2016). Description of “Candidatus Marispirochaeta associata” and reclassification of Spirochaeta bajacaliforniensis, Spirochaeta smaragdinae and Spirochaeta sinaica to a new genus Sediminispirochaeta gen. Nov. As Sediminispirochaeta bajacaliforniensis comb. Nov., Sediminispirochaeta smaragdinae comb. Nov. And Sediminispirochaeta sinaica comb. Nov. *International Journal of Systematic and Evolutionary Microbiology*, *66*(12), 5485–5492. https://doi.org/10.1099/ijsem.0.001545

Suarez, C., Ratering, S., Geissler-Plaum, R., & Schnell, S. (2014). Hartmannibacter diazotrophicus gen. Nov., sp. Nov., a phosphate-solubilizing and nitrogen-fixing alphaproteobacterium isolated from the rhizosphere of a natural salt-meadow plant. *International Journal of Systematic and Evolutionary Microbiology*, *64*(Pt 9), 3160–3167. https://doi.org/10.1099/ijs.0.064154-0

Suárez-Moo, P., Cruz-Rosales, M., Ibarra-Laclette, E., Desgarennes, D., Huerta, C., & Lamelas, A. (2020). Diversity and Composition of the Gut Microbiota in the Developmental Stages of the Dung Beetle Copris incertus Say (Coleoptera, Scarabaeidae). *Frontiers in Microbiology*, *11*. https://doi.org/10.3389/fmicb.2020.01698

Tavormina, P. L., Hatzenpichler, R., McGlynn, S., Chadwick, G., Dawson, K. S., Connon, S. A., & Orphan, V. J. (2015). Methyloprofundus sedimenti gen. Nov., sp. Nov., an obligate methanotroph from ocean sediment belonging to the “deep sea-1” clade of marine methanotrophs. *International Journal of Systematic and Evolutionary Microbiology*, *65*(Pt 1), 251–259. https://doi.org/10.1099/ijs.0.062927-0

Thajudeen, J., Yousuf, J., Veetil, V. P., Varghese, S., Singh, A., & Abdulla, M. H. (2017). Nitrogen fixing bacterial diversity in a tropical estuarine sediments. *World Journal of Microbiology & Biotechnology*, *33*(2), 41. https://doi.org/10.1007/s11274-017-2205-x

Ullrich, S. R., González, C., Poehlein, A., Tischler, J. S., Daniel, R., Schlömann, M., Holmes, D. S., & Mühling, M. (2016). Gene Loss and Horizontal Gene Transfer Contributed to the Genome Evolution of the Extreme Acidophile “Ferrovum.” *Frontiers in Microbiology*, *7*, 797. https://doi.org/10.3389/fmicb.2016.00797

Vera-Ponce de León, A., Ormeño-Orrillo, E., Ramírez-Puebla, S. T., Rosenblueth, M., Degli Esposti, M., Martínez-Romero, J., & Martínez-Romero, E. (2017). Candidatus Dactylopiibacterium carminicum, a Nitrogen-Fixing Symbiont of Dactylopius Cochineal Insects (Hemiptera: Coccoidea: Dactylopiidae). *Genome Biology and Evolution*, *9*(9), 2237–2250. https://doi.org/10.1093/gbe/evx156

Vos, P., Garrity, G., Jones, D., Krieg, N. R., Ludwig, W., Rainey, F. A., Schleifer, K.-H., & Whitman, W. B. (2011). *Bergey’s Manual of Systematic Bacteriology: Volume 3: The Firmicutes*. Springer Science & Business Media.

Watanabe, A., & Yamamoto, Y. (1967). Heterotrophic Nitrogen Fixation by the Blue-Green Alga Anabaenopsis circularis. *Nature*, *214*(5089), 738–738. https://doi.org/10.1038/214738a0

Willis, A., Chuang, A. W., & Burford, M. A. (2016). Nitrogen fixation by the diazotroph Cylindrospermopsis raciborskii (Cyanophyceae). *Journal of Phycology*, *52*(5), 854–862. https://doi.org/10.1111/jpy.12451

Zavarzina, D. G., Kolganova, T. V., Bulygina, E. S., Kostrikina, N. A., Turova, T. P., & Zavarzin, G. A. (2006). [Geoalkalibacter ferrihydriticus gen. Nov., sp. Nov., the first alkaliphilic representative of the family Geobacteraceae, isolated from a soda lake]. *Mikrobiologiia*, *75*(6), 775–785.

Zavarzina, D. G., Tourova, T. P., Kolganova, T. V., Boulygina, E. S., & Zhilina, T. N. (2009). Description of Anaerobacillus alkalilacustre gen. Nov., sp. Nov.—Strictly anaerobic diazotrophic bacillus isolated from soda lake and transfer of Bacillus arseniciselenatis, Bacillus macyae, and Bacillus alkalidiazotrophicus to Anaerobacillus as the new combinations A. arseniciselenatis comb. Nov., A. macyae comb. Nov., and A. alkalidiazotrophicus comb. Nov. *Microbiology*, *78*(6), 723–731. https://doi.org/10.1134/S0026261709060095

Zehr, J. P., Mellon, M. T., & Hiorns, W. D. (1997). Phylogeny of cyanobacterial nifH genes: Evolutionary implications and potential applications to natural assemblages. *Microbiology*, *143*(4), 1443–1450. https://doi.org/10.1099/00221287-143-4-1443

Zhang, Z., Li, D., Shi, X., Zhai, Y., Guo, Y., Zheng, Y., Zhao, L., He, Y., Chen, Y., Wang, Z., Su, J., Kang, Y., & Gao, Z. (2020). Genomic characterization of an emerging Enterobacteriaceae species: The first case of co-infection with a typical pathogen in a human patient. *BMC Genomics*, *21*(1), 297. https://doi.org/10.1186/s12864-020-6720-z

정영륜, 비비페미다, 정유진, & 김근곤. (2013). *Martelella endophytica yc6887 microbial strain having a plant-pathology biocontrol effect and nitrogen-fixing effect, multifunctional microorganism preparation comprising same, and gene base sequence having a nitrogen-fixing effect derived therefrom* (World Intellectual Property Organization Patent No. WO2013008974A1). https://patents.google.com/patent/WO2013008974A1/en
