# Supplementary material for: Phylogeny of Nitrogenase Structural and Assembly Components Reveals New Insights into the Origin and Distribution of Nitrogen Fixation across Bacteria and Archaea
Source: Microorganisms. 2021 Aug 4;9(8):1662. doi: 10.3390/microorganisms9081662 (PMC8399215; doi:10.3390/microorganisms9081662)
Supplement: Supplementary file 1 [file microorganisms-09-01662-s001.zip › Archive 2/Supplementary Figures.docx]

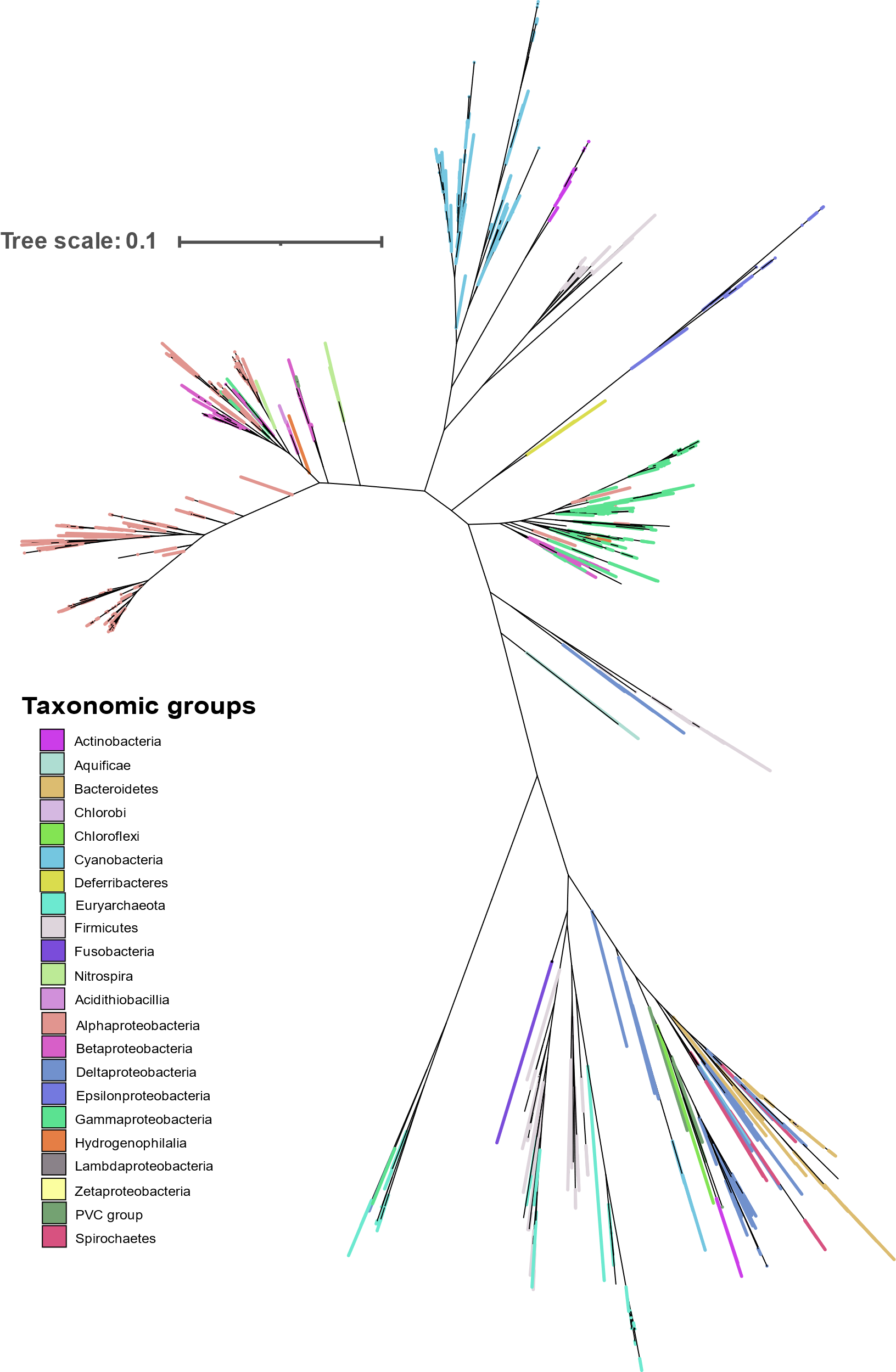


Figure S1: Phylogenetic analysis of individual NifH proteins by FastTree using the JTT+CAT evolution model.


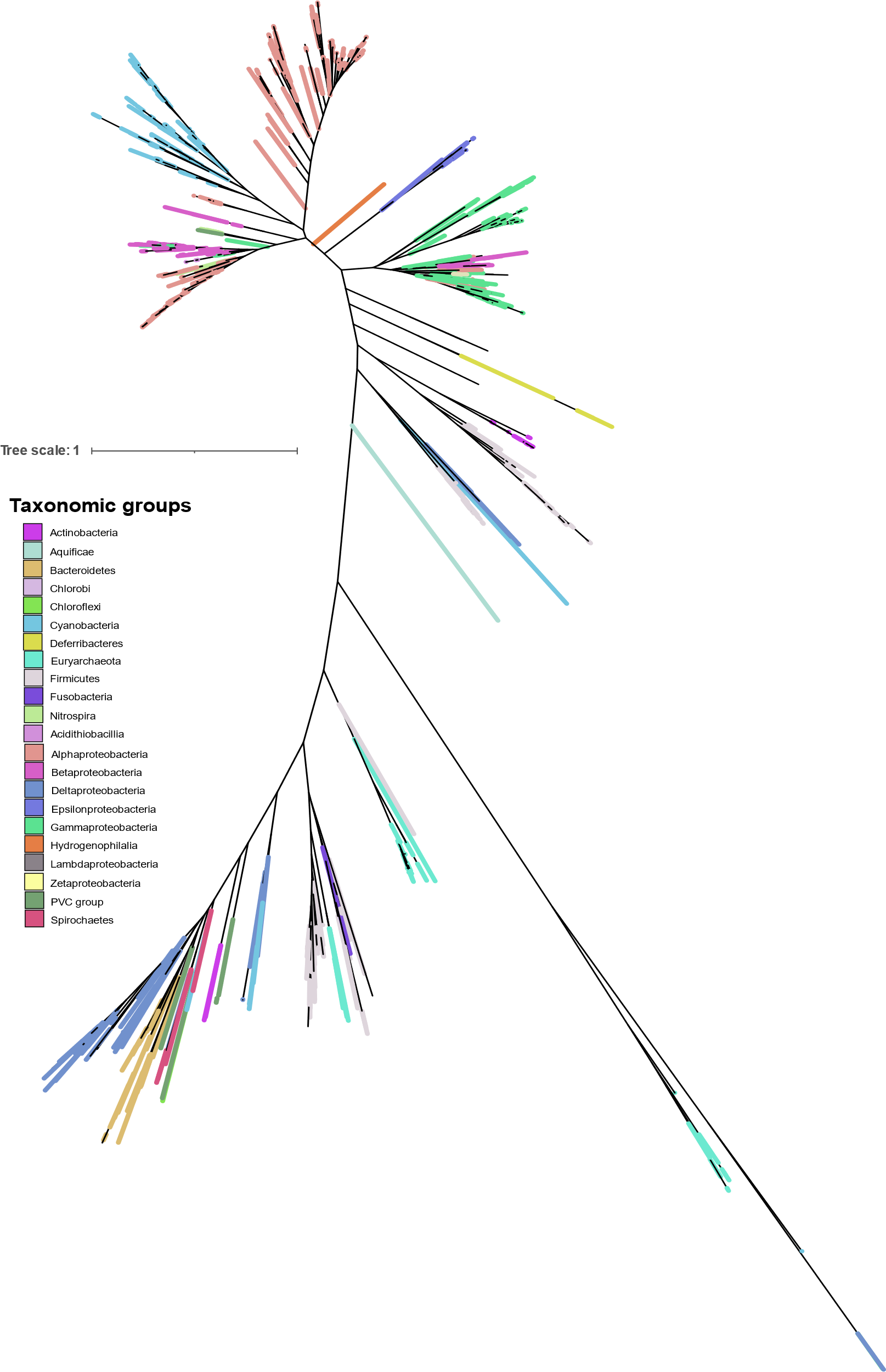


Figure S2: Phylogenetic analysis of individual NifD proteins by FastTree using the JTT+CAT evolution model.


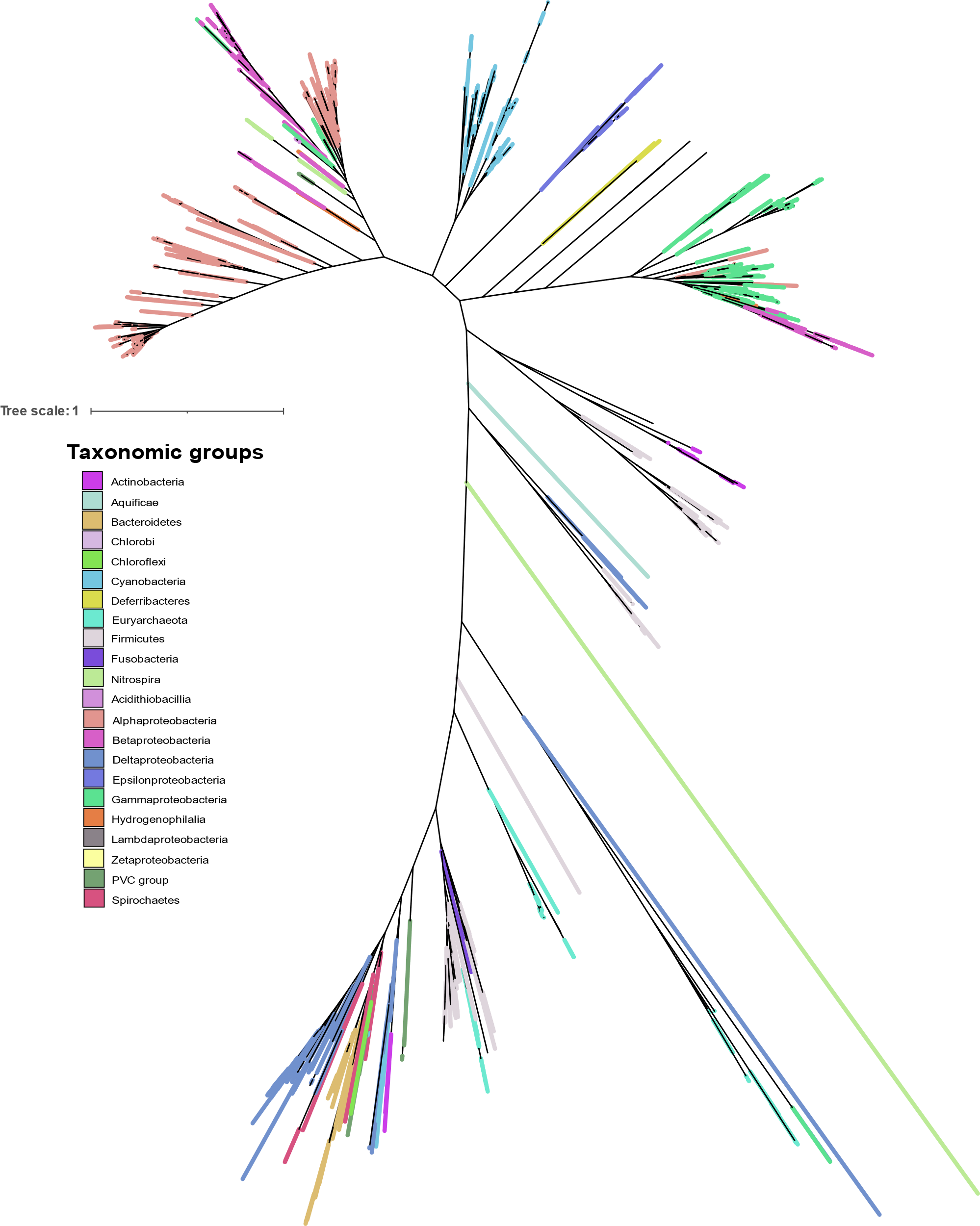


Figure S3: Phylogenetic analysis of individual NifK proteins by FastTree using the JTT+CAT evolution model.


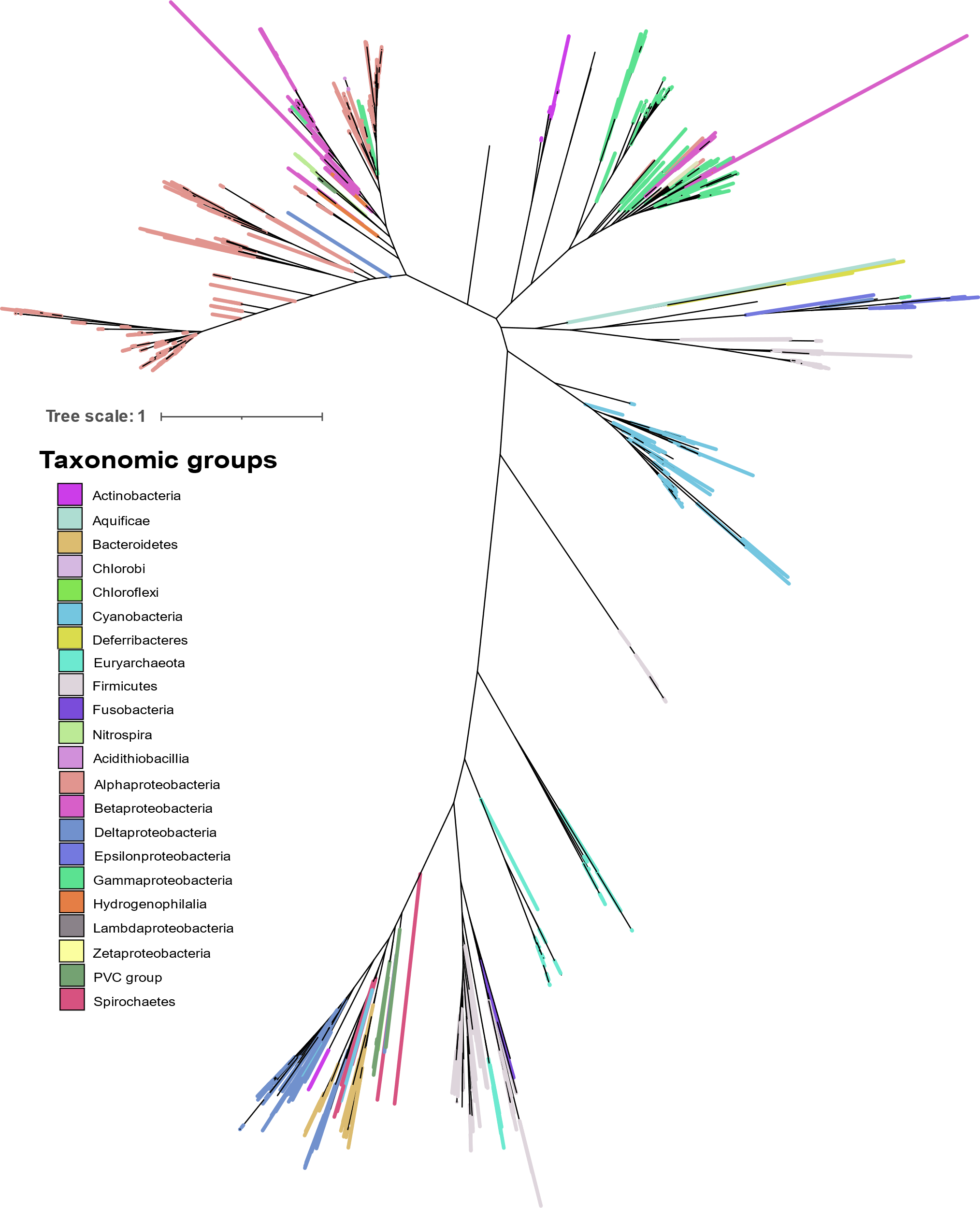


Figure S4: Phylogenetic analysis of individual NifE proteins by FastTree using the JTT+CAT evolution model.


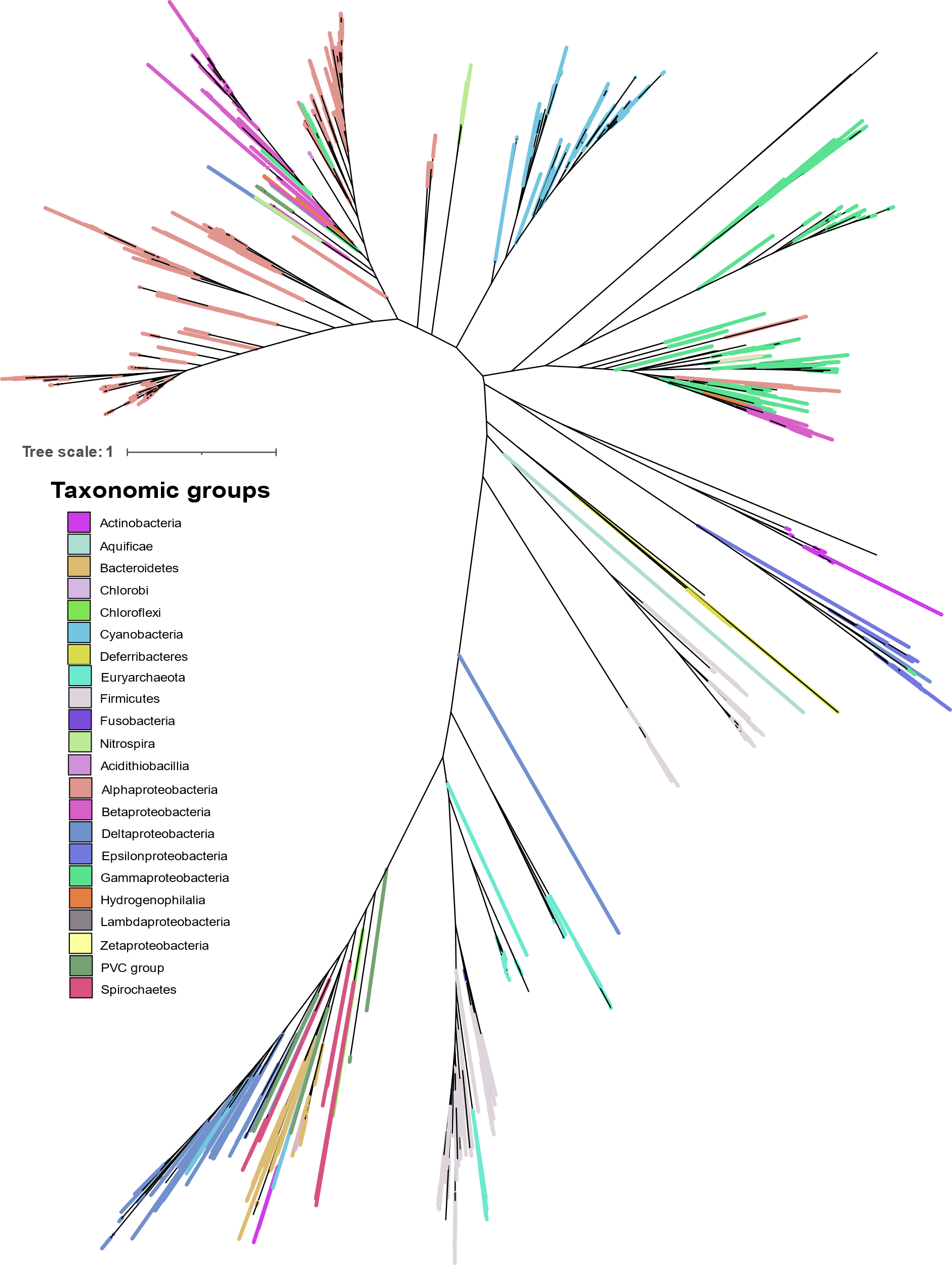


Figure S5: Phylogenetic analysis of individual NifN proteins by FastTree using the JTT+CAT evolution model.


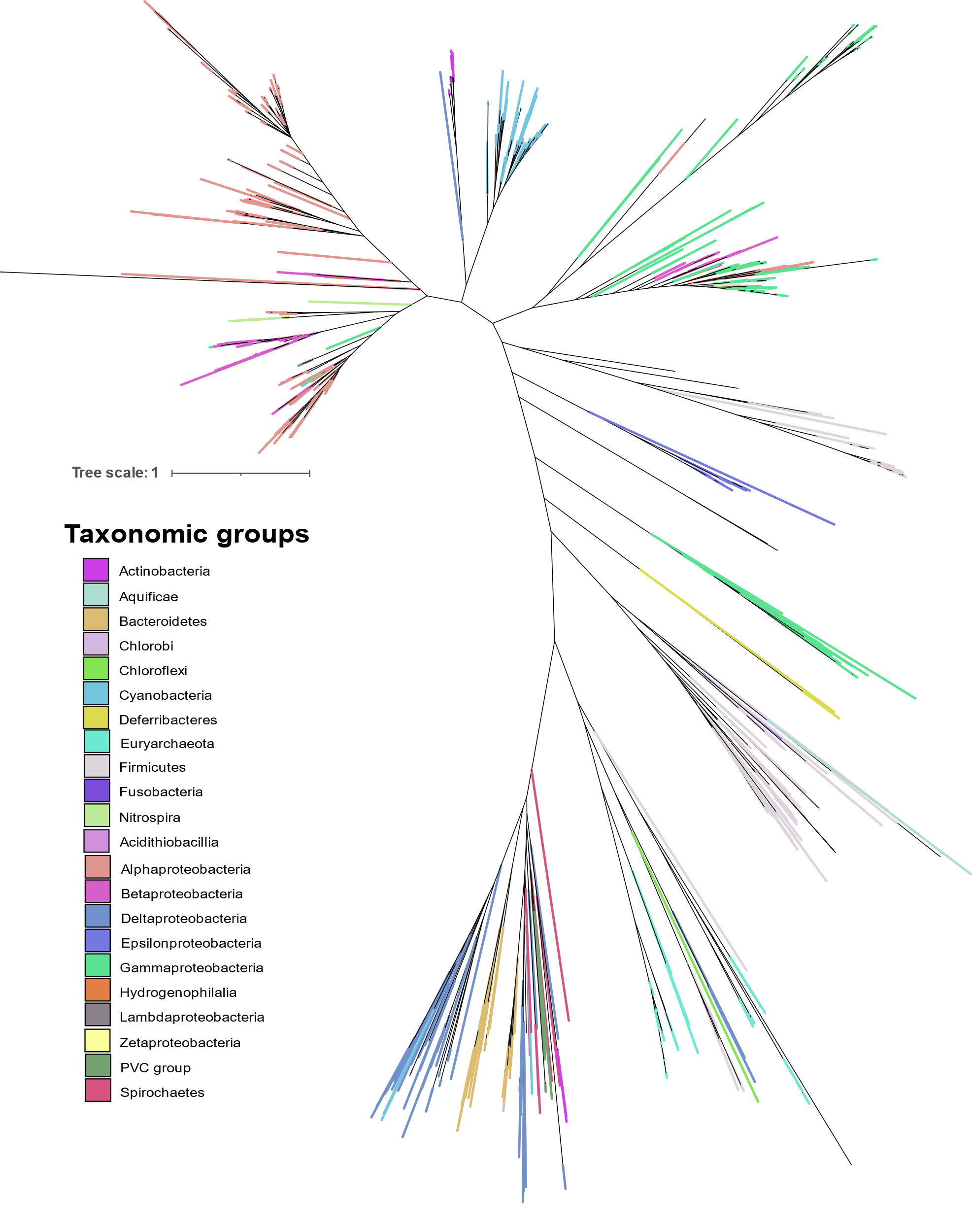


Figure S6: Phylogenetic analysis of individual NifN proteins by FastTree using the JTT+CAT evolution model.


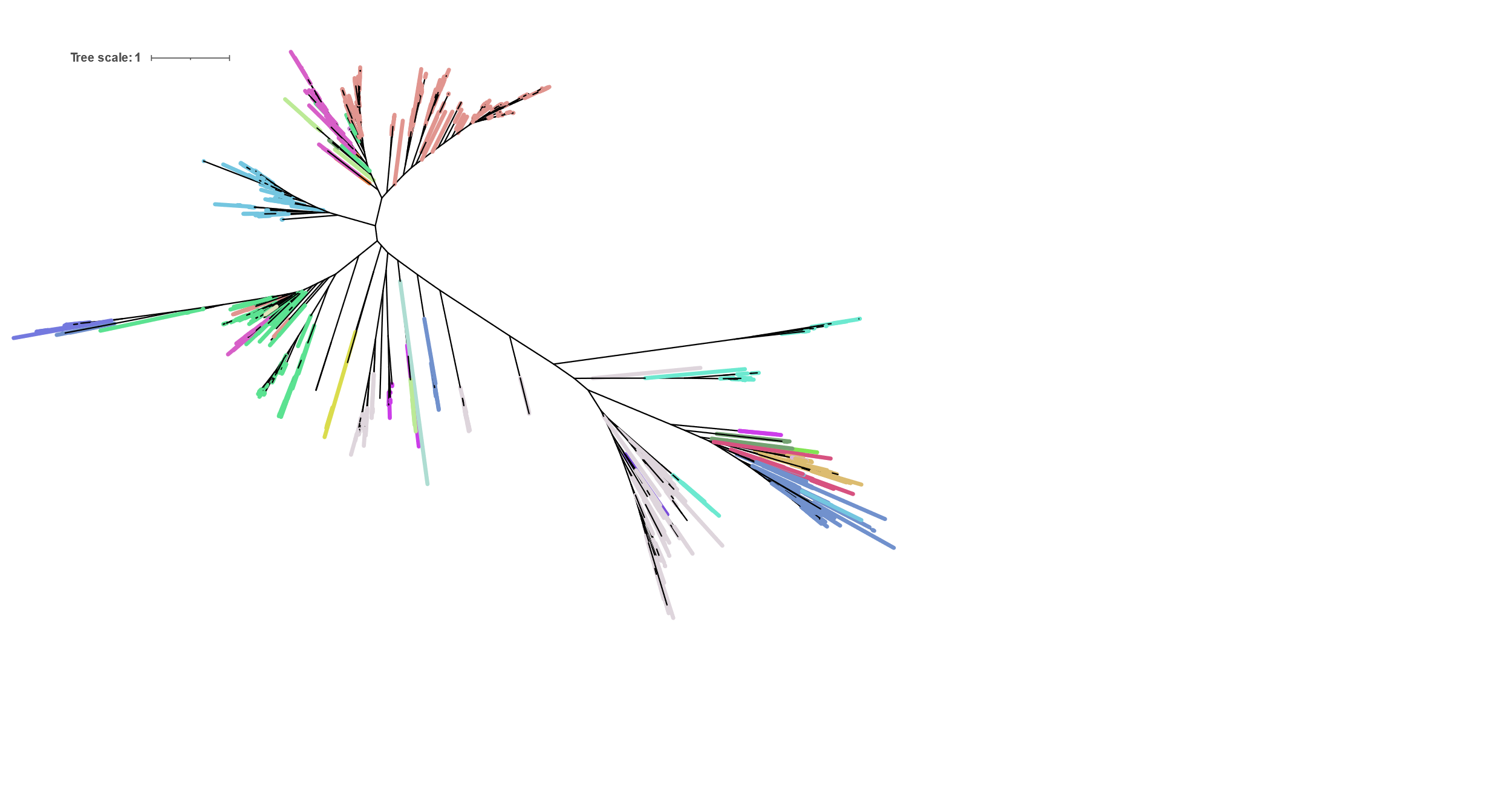


Figure S7: Molecular phylogenetic analysis of concatenated NifHDKENB proteins by Maximum Likelihood (PhyML) with branch support by posterior probability. Each clade is highlighted by the bacterial or archaeal phylum and Protebacteria are further divided into classes.


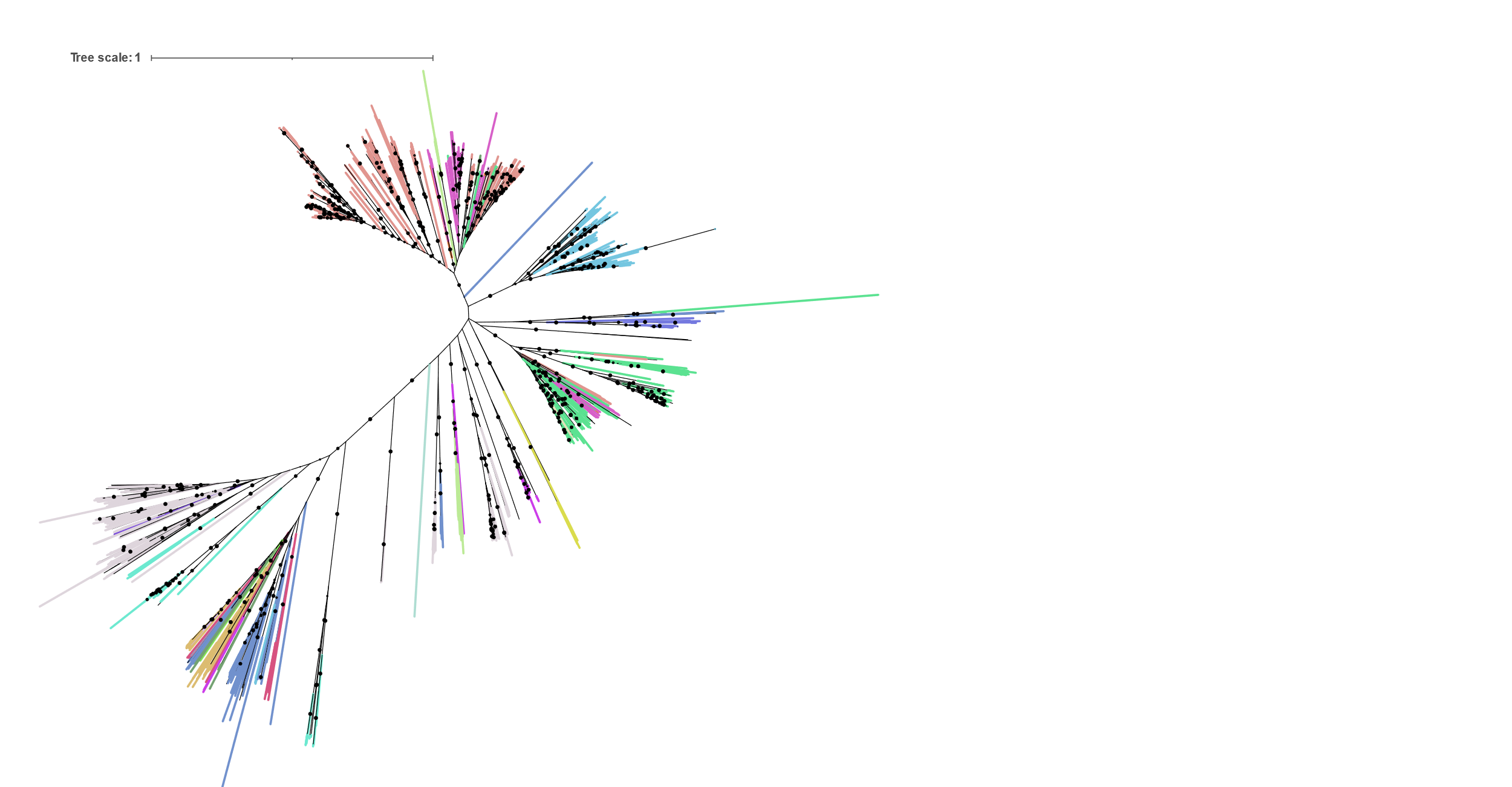


Figure S8: Molecular phylogenetic analysis of concatenated NifHDKENB proteins Neighbour joining by RapidNJ using Kimura model. Each clade is highlighted by the bacterial or archaeal phylum and Protebacteria are further divided into classes.


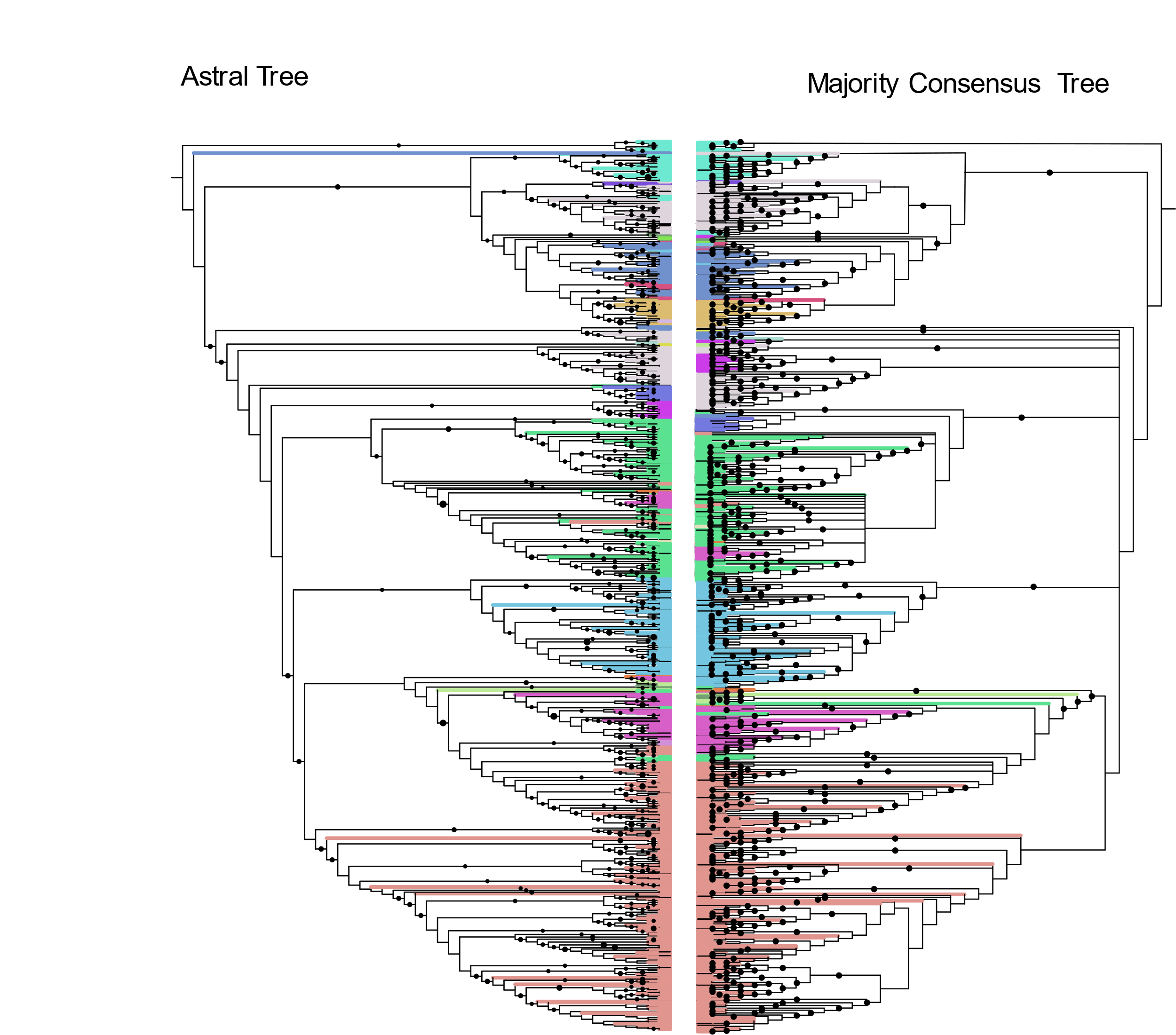


Figure S9: Cladogram showing comparison of Astral tree obtained from six individual trees Vs majority consensus tree obtained from three trees obtained by FASTTREE, PhyML, and Neighbour Joining using concatenated NifHDKENB proteins. Black dots in Astral tree represents the Astral bootstrapping and on the concatenated tree represents the branching present in all three trees and all other splits present in at least two trees.


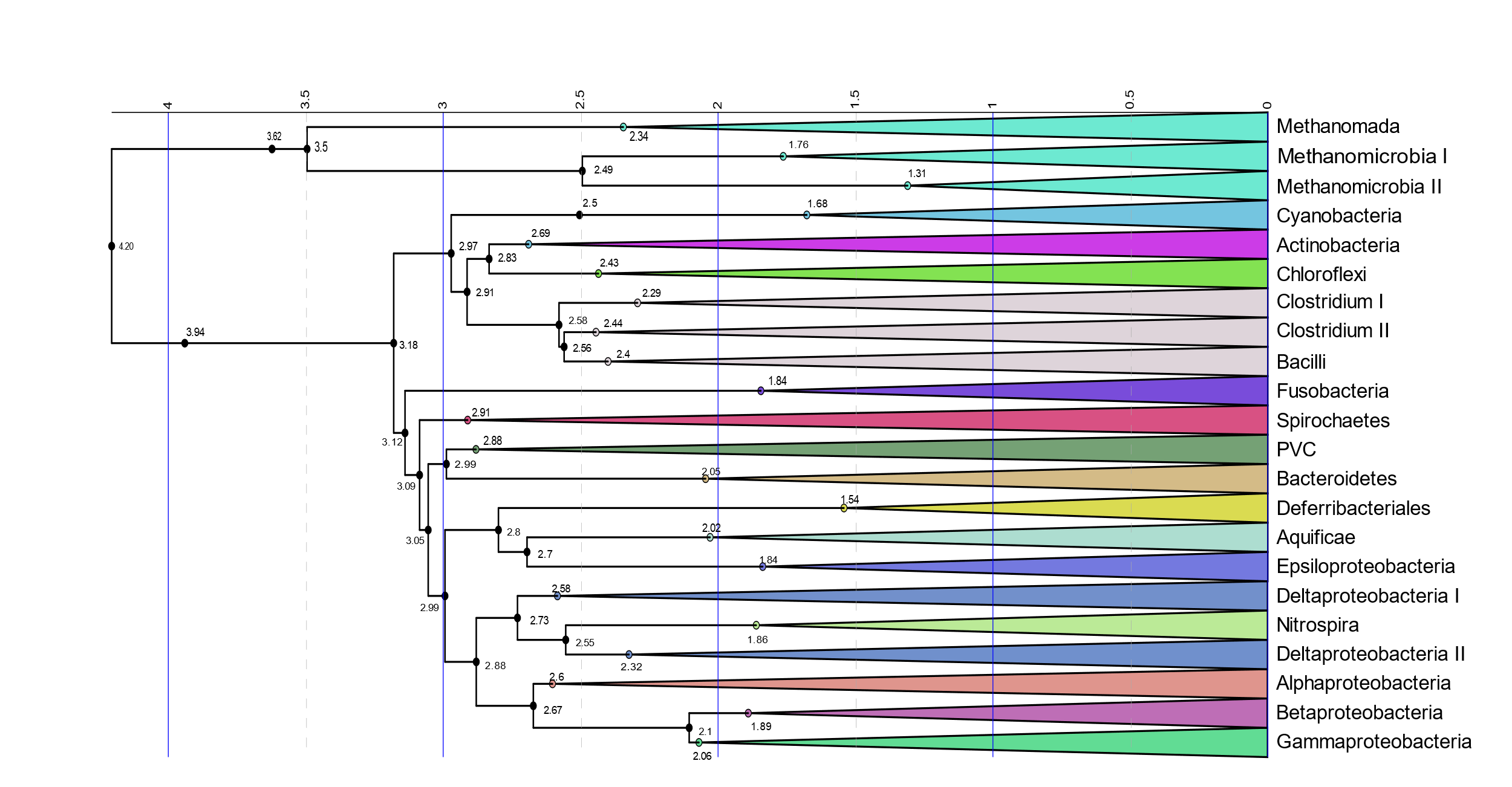


Figure S10: Chronogram of evolution of diazotrophs obtained by selecting diazotrophic genera from the microbial evolution tree proposed by Zhu et al 2019 using source data file. Node labels represent the time in billion years ago (Ga) in the original tree.


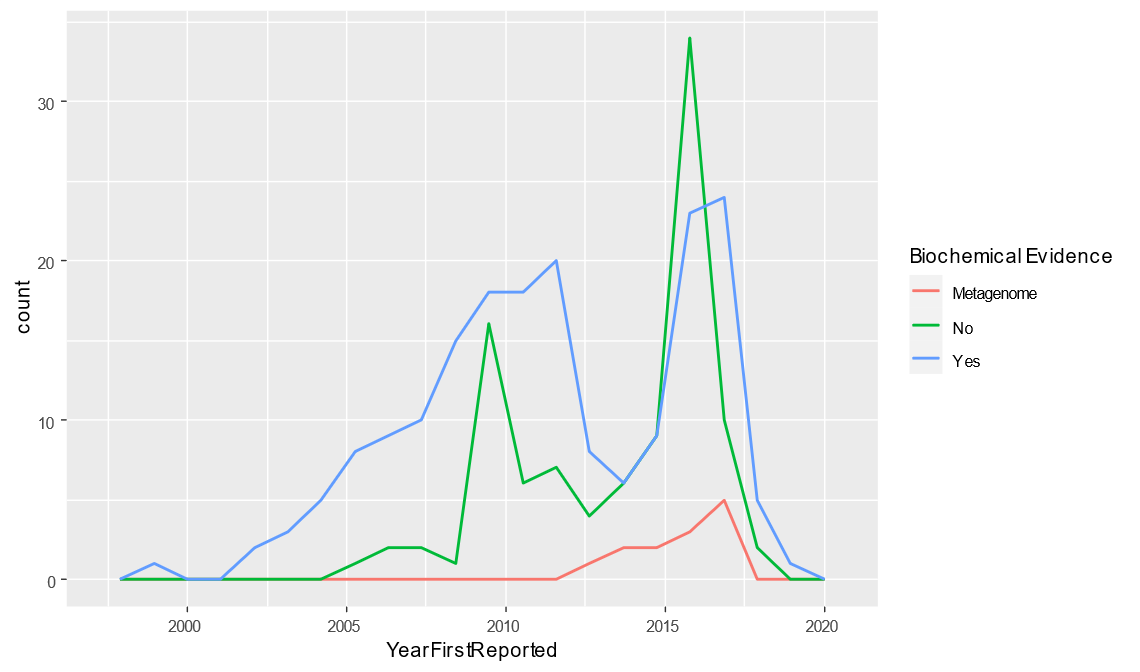


Figure S11: Number of genomes containing all six *nifHDKENB* genes according to the year they were reported. Number of genomes without biochemical evidence increased rapidly with the increase in the number of genomes reported.
