# Supplementary figures and images for: Phylogeny of Nitrogenase Structural and Assembly Components Reveals New Insights into the Origin and Distribution of Nitrogen Fixation across Bacteria and Archaea
Source: Microorganisms. 2021 Aug 4;9(8):1662. doi: 10.3390/microorganisms9081662 (PMC8399215; doi:10.3390/microorganisms9081662)

Tree scale: 1

NifHDKENB

16S rRNA

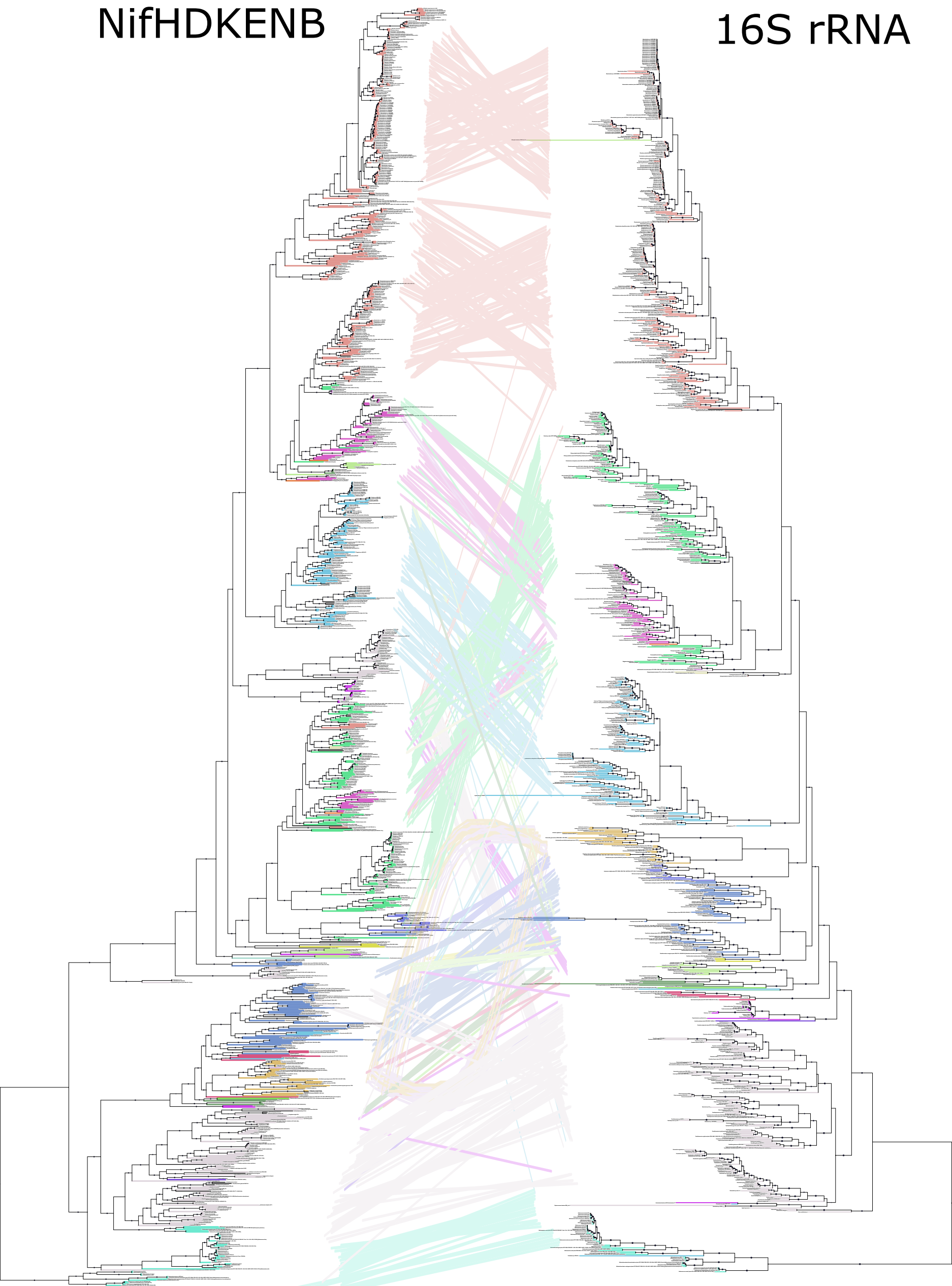

Supplement: Supplementary file 1 [file microorganisms-09-01662-s001.zip › Archive 2/Supplementary tree file.pdf]
